# Posttranslational regulation of photosynthetic activity via the TOR kinase in plants

**DOI:** 10.1126/sciadv.adj3268

**Published:** 2024-06-19

**Authors:** Stefano D’Alessandro, Florent Velay, Régine Lebrun, Delyan Zafirov, Marwa Mehrez, Shanna Romand, Rim Saadouni, Céline Forzani, Sylvie Citerne, Marie-Hélène Montané, Christophe Robaglia, Benoît Menand, Christian Meyer, Ben Field

**Affiliations:** ^1^ Aix Marseille Univ, CEA, CNRS, BIAM, LGBP Team, 13009 Marseille, France.; ^2^ Università di Torino, Dipartimento di Scienze della vita e Biologia dei Sistemi, 10135 Torino, Italy.; ^3^ Aix Marseille Univ, CNRS, Plate-forme Protéomique, Marseille Protéomique (MaP), IMM FR 3479, 31 Chemin Joseph Aiguier, 13009 Marseille, France.; ^4^ Faculty of Sciences of Tunis, University of Tunis El Manar, 2092 Tunis, Tunisia.; ^5^ Institut Jean-Pierre Bourgin, INRAE, AgroParisTech, CNRS, Université Paris-Saclay, 78000 Versailles, France.

## Abstract

Chloroplasts are the powerhouse of the plant cell, and their activity must be matched to plant growth to avoid photooxidative damage. We have identified a posttranslational mechanism linking the eukaryotic target of rapamycin (TOR) kinase that promotes growth and the guanosine tetraphosphate (ppGpp) signaling pathway of prokaryotic origins that regulates chloroplast activity and photosynthesis in particular. We find that RelA SpoT homolog 3 (RSH3), a nuclear-encoded enzyme responsible for ppGpp biosynthesis, interacts directly with the TOR complex via a plant-specific amino-terminal region which is phosphorylated in a TOR-dependent manner. Down-regulating TOR activity causes a rapid increase in ppGpp synthesis in RSH3 overexpressors and reduces photosynthetic capacity in an RSH-dependent manner in wild-type plants. The TOR-RSH3 signaling axis therefore regulates the equilibrium between chloroplast activity and plant growth, setting a precedent for the regulation of organellar function by TOR.

## Teaser

The eukaryotic TOR kinase regulates plant photosynthesis by posttranslational modulation of guanosine tetraphosphate signaling.

## INTRODUCTION

The use of sunlight to fix carbon and produce chemical energy during photosynthesis is the basis of almost all life on the planet. However, the photosynthetic machinery is also resource intensive, and chloroplasts must be tightly regulated to prevent photooxidative stress. Guanosine tetraphosphate (ppGpp) is a signaling nucleotide synthesized by RelA SpoT homolog (RSH) enzymes that regulate growth and stress acclimation in the majority of prokaryotes (*1*). In plants, ppGpp negatively regulates photosynthesis and is required for normal growth and stress acclimation (*2*–*4*). Target of rapamycin (TOR) is a nucleocytosolic Ser/Thr kinase complex that plays an evolutionary conserved role in eukaryotes by promoting growth in response to favorable environmental cues (*5*, *6*). Nutrient limitation or environmental stress leads to the inactivation of TOR, which slows growth and promotes nutrient recycling. TOR also influences photosynthesis in plants and algae, a phenomenon that up to now was principally explained by transcriptional regulation of nuclear-encoded chloroplast genes (*7*–*11*). The possible posttranslational regulation of photosynthesis by TOR was recently proposed (*12*), although it is unclear whether it is direct, and a specific mechanism has yet to be identified. Here, we set out to determine whether TOR is involved in the direct posttranscriptional regulation of photosynthesis.

## RESULTS

### A subunit of the TOR complex interacts with RSH enzymes for ppGpp metabolism

**Fig. 1.**
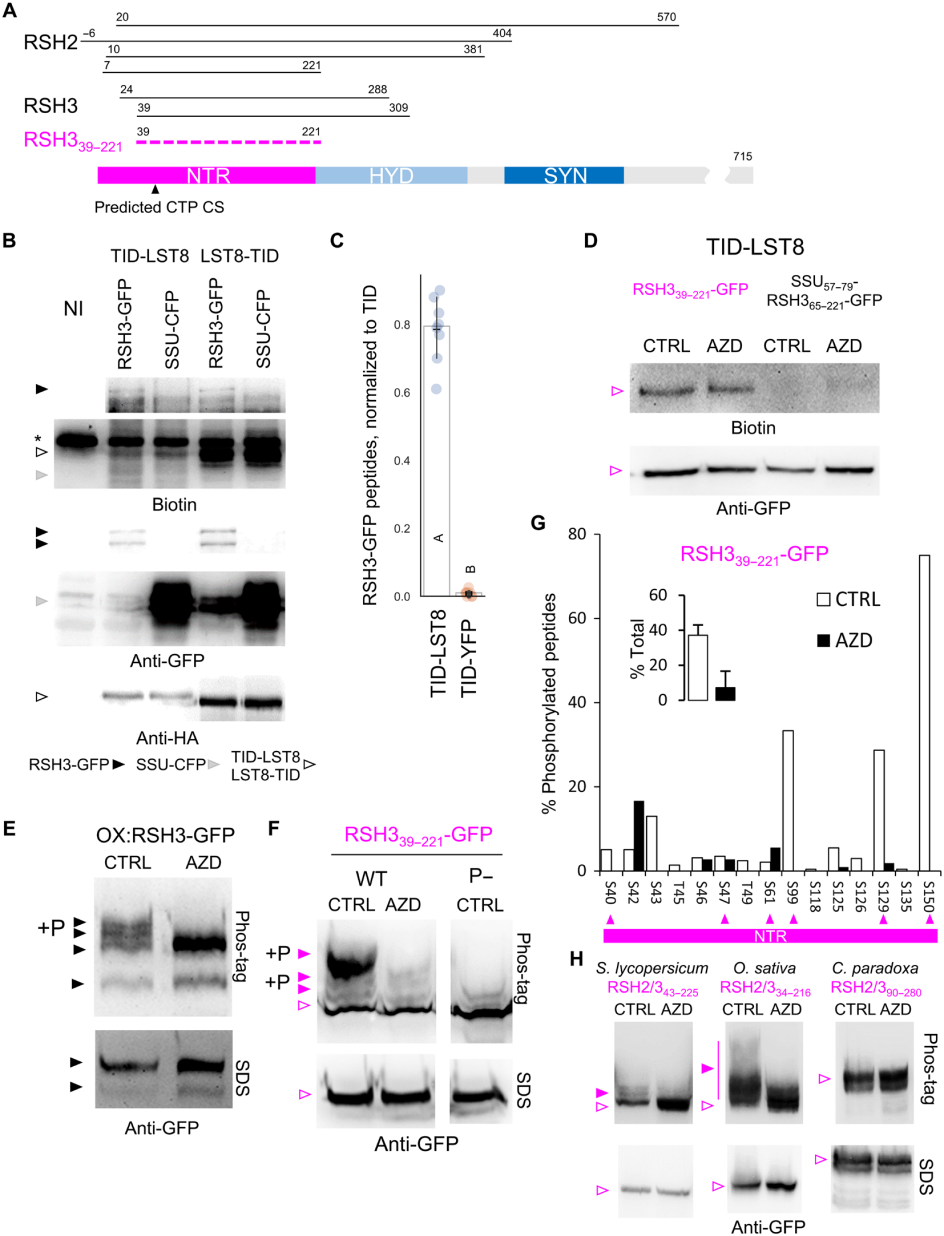
RSH enzymes interact with a subunit of the TOR complex and undergo TOR-dependent phosphorylation. (**A**) Alignment of the Y2H LST8 interacting regions of RSH2/3, showing the minimal interaction zone (magenta dashed line). TargetP predicted CTP cleavage site (CS) shown. NTR, N-terminal region; SYN, synthetase domain; HYD, hydrolase domain. (**B**) A proximity labeling experiment showing selected regions from blots of protein extracts from *N. benthamiana* coexpressing TID LST8 fusions with RSH3-GFP or SSU-CFP. Leaves were infiltrated with biotin for 2 hours. NI, non-inoculated control; asterisk (*) represents endogenous biotinylated protein. (**C**) Liquid chromatography–mass spectrometry (LC-MS) identification of RSH3-GFP peptides in the biotinylated protein fraction from *N. benthamiana* coexpressing RSH3-GFP with TID-LST8 or TID-YFP. Blots of protein extracts from (**D**) *N. benthamiana* coexpressing TID-LST8 and RSH3_39–221_-GFP or SSU_57–79_-RSH_365–221_-GFP, (**E**) Arabidopsis OX:RSH3-GFP seedlings, and (**F**) *N. benthamiana* expressing RSH3_39–221_-GFP or a phosphodefective (P−) form and treated with AZD-8055 (AZD) or the mock control (CTRL). Arabidopsis seedlings were sampled 48 hours after treatment and *N. benthamiana* at 2 hours. Target protein phosphoforms are indicated by P+. (**G**) Map of RSH3_39–221_-GFP phosphorylation sites identified by LC-MS following immunoprecipitation from *N. benthamiana*. Arrowheads indicate phosphorylation in putative TOR-dependent contexts (see also fig. S6). (**H**) Blots of protein extracts from *N. benthamiana* expressing RSH3_39–221_ homologous regions from *Solanum lycopersicum*, *Oryza sativa*, and *C. paradoxa* treated with AZD-8055 (AZD) or the mock control (CTRL) for 2 hours. Filled arrowheads indicate target protein phosphoforms. SDS, SDS-PAGE separation; phos-tag, phos-tag SDS-PAGE separation. Full blots and source data are available in data S1. WT, wild type.

Using the TOR complex subunit lethal with SEC13 8 (LST8) as bait in an untargeted yeast two-hybrid (Y2H) screen, we identified the bifunctional ppGpp synthase/hydrolase enzymes RSH2 and RSH3 as LST8 interactors (Fig. 1A and table S1). RSH2 and RSH3 are closely related paralogs (85% similarity) from the conserved RSH2/3 family that play an important role in ppGpp metabolism during development and stress acclimation (*4*). LST8 is nucleocytosolic (fig. S1), while RSH2 and RSH3 are nuclear-encoded chloroplast enzymes expected to reside only transiently in the cytosol before chloroplast import and processing (*13*, *14*). Therefore, we adopted a proximity labeling approach to determine whether LST8 interacts with the RSH3 precursor protein in planta. LST8 fused to the promiscuous biotin ligase TurboID (*15*) with a triple hemagglutinin (HA) tag (TID-LST8 and LST8-TID) was coexpressed with RSH3–green fluorescent protein (GFP) or cyan fluorescent protein targeted to the chloroplast by the Rubisco small subunit 5A chloroplast transit peptide (CTP) (SSU-CFP). Upon expression of TID-LST8 or LST8-TID, we observed biotinylated proteins at the molecular weight of RSH3-GFP only in the samples coexpressing RSH3-GFP and not in the SSU-CFP control (Fig. 1B). SSU-CFP (Fig. 1B, gray arrowheads) and SSU-GFP (fig. S2) are not biotinylated by TID-LST8, indicating that RSH3-GFP biotinylation is specific. We further confirmed the specificity of TID-LST8 biotinylation by purifying biotinylated proteins and showing a strong enrichment for RSH3-GFP peptides compared to a TID-YFP control (Fig. 1C).

We next sought to determine whether RSH3_39–221_, the minimal LST8 interaction zone defined by six RSH2/3 Y2H clones (Fig. 1A), was sufficient for interaction with LST8 in planta. RSH3_39–221_-GFP was strongly biotinylated by TID-LST8 (Fig. 1D). A modified control protein SSU_59–75_-RSH3_65–221_-GFP, where the region corresponding to the predicted CTP was substituted with an equivalent region of the SSU CTP (fig. S3A), was not biotinylated by TID-LST8 despite accumulating to the same level in the cytosol. Biotinylation was also not affected by inhibition of TOR with AZD-8055 (AZD), an adenosine triphosphate (ATP)–competitive inhibitor selective for TOR (*16*). The RSH3_39–221_ region is therefore sufficient for interaction with LST8 in planta, and residues 39 to 64 are required.

### The RSH3 NTR determines RSH3 stability and localization

We observed that RSH3-GFP accumulates to very low levels in planta (Fig. 1B and fig. S4A), shows dual bands by immunoblot (Fig. 1, B and E, and fig. S4A), and only has sporadic chloroplast localization (fig. S4B). To determine whether the RSH3 N-terminal region (NTR) regulates RSH3 localization and stability, we substituted the predicted CTP with the SSU CTP while either preserving the remaining NTR (SSU-RSH3_65-END_-GFP) or eliminating the majority of the NTR (SSU-RSH3*_195-END_-GFP) (fig. S3B). Replacement of the predicted CTP alone did not cause a major change in protein localization (fig. S4B). However, elimination of the NTR resulted in strong accumulation of the mature form of RSH3 in the chloroplast (fig. S4, A and B). Therefore, the full NTR is involved in controlling RSH3 accumulation and localization.

We then found that the RSH3 CTP is much longer than predicted. We observed that the probable mature forms of RSH3 were of a similar size whether the NTR was present or not (fig. S4B). This suggested the presence of additional downstream processing sites. Analysis of a series of N-terminal GFP fusions (fig. S3C) showed that RSH3_1–165_-GFP was the first to show clear chloroplast localization and processing (fig. S5, A and B). The mature form was similar in size to GFP, suggesting that the chloroplast cleavage site was close to position 165 (fig. S5A). Accumulation of a larger mature protein demonstrated that this cleavage site was also retained in RSH3_1–175_-GFP. These observations are consistent with the increased probability of cleavage between 160 and 161 (fig. S5C). The dual chloroplast and nucleocytoplasmic localization of RSH3_1–175_-GFP strongly resembled that of full-length RSH3, and there was reduced accumulation compared to RSH3_1–165_-GFP. Motifs important for destabilization may therefore lie between RSH3 positions 165 and 175. In conclusion, the RSH3 NTR contains a remarkably long CTP, more than double the average (*17*), and is responsible for destabilizing RSH3 and conferring a dual nucleocytosolic and chloroplastic localization.

### The RSH3 NTR is phosphorylated in a TOR-dependent manner

We next tested whether the TOR complex is involved in phosphorylation of RSH3. We observed phosphoforms of the RSH3-GFP precursor in Arabidopsis OX:RSH3-GFP plants (Fig. 1E). Notably, the phosphoforms were lost rapidly upon TOR inhibition. We did not observe phosphoforms for mature RSH3-GFP (Fig. 1E, lower band), suggesting that TOR-dependent phosphorylation occurs only on the CTP which is subsequently cleaved and degraded after chloroplast import.

We then analyzed RSH3_39–221_ and found serine residues in TOR phosphorylation–compatible contexts (fig. S6A) (*18*, *19*). When expressed in *Nicotiana benthamiana*, several RSH3_39–221_-GFP phosphoforms accumulated under control conditions and disappeared upon TOR inhibition (Fig. 1F). Mutation of TOR-compatible phosphosites in RSH3_39–221_-GFP almost completely abolished TOR-dependent phosphorylation. Liquid chromatography–mass spectrometry (LC-MS) analysis of immunoprecipitated RSH3_39–221_-GFP identified phosphorylation at 15 serine residues, including five putative TOR-dependent phosphosites (Fig. 1G). TOR inhibition caused a marked reduction in the proportion of phosphorylated peptides, as expected. Together, these results indicate that the RSH3 CTP is hyperphosphorylated in a TOR-dependent manner and that hyperphosphorylation requires serine residues in canonical TOR-dependent contexts.

### Evolution of the RSH2/3 family NTR

At least three conserved families of RSH enzymes with distinct evolutionary origins can be identified in plants and algae: RSH1, RSH2/3, and RSH4 (*20*, *21*). We found that regions homologous to the minimal LST8 interaction zone of RSH3 are found only in the NTR of RSH2/3 family members in plants and algae and are absent from RSH1 and RSH4/CRSH family enzymes, as well as from prokaryotic RSH enzymes (fig. S6A). The glaucophyte algae *Cyanophora paradoxa* RSH2/3 enzyme has an NTR-like region (fig. S6B), indicating possible origins before the divergence of green algae and land plants from glaucophytes, almost 2 billion years ago (*22*). Two of the TOR-dependent phosphosites identified on RSH3 (S99 and S129) are conserved on the *C. paradoxa* NTR, and there are six serine-proline (SP) motifs, which are favored sites for TOR-dependent phosphorylation (*19*). RSH2/3 family NTRs tend to have multiple SP motifs, while the unrelated NTR of RSH1 has none. Another characteristic of RSH2/3 family NTRs is the presence of a long intrinsically disordered region that is absent from the RSH1 NTR (fig. S7).

We next tested selected RSH2/3 NTRs in planta to determine whether TOR-dependent phosphorylation is widely conserved. We found that tomato and rice RSH2/3 NTRs were phosphorylated in a TOR-dependent manner (Fig. 1H). The *C. paradoxa* RSH2/3 family NTR was not phosphorylated, suggesting either divergent TOR recognition or a later emergence of TOR-dependent regulation. Together, we show that the RSH3 NTR has ancient evolutionary origins and that phosphorylation by TOR is conserved among members of the RSH2/3 family in at least the flowering plants.

### TOR down-regulates photosynthesis via ppGpp signaling

**Fig. 2.**
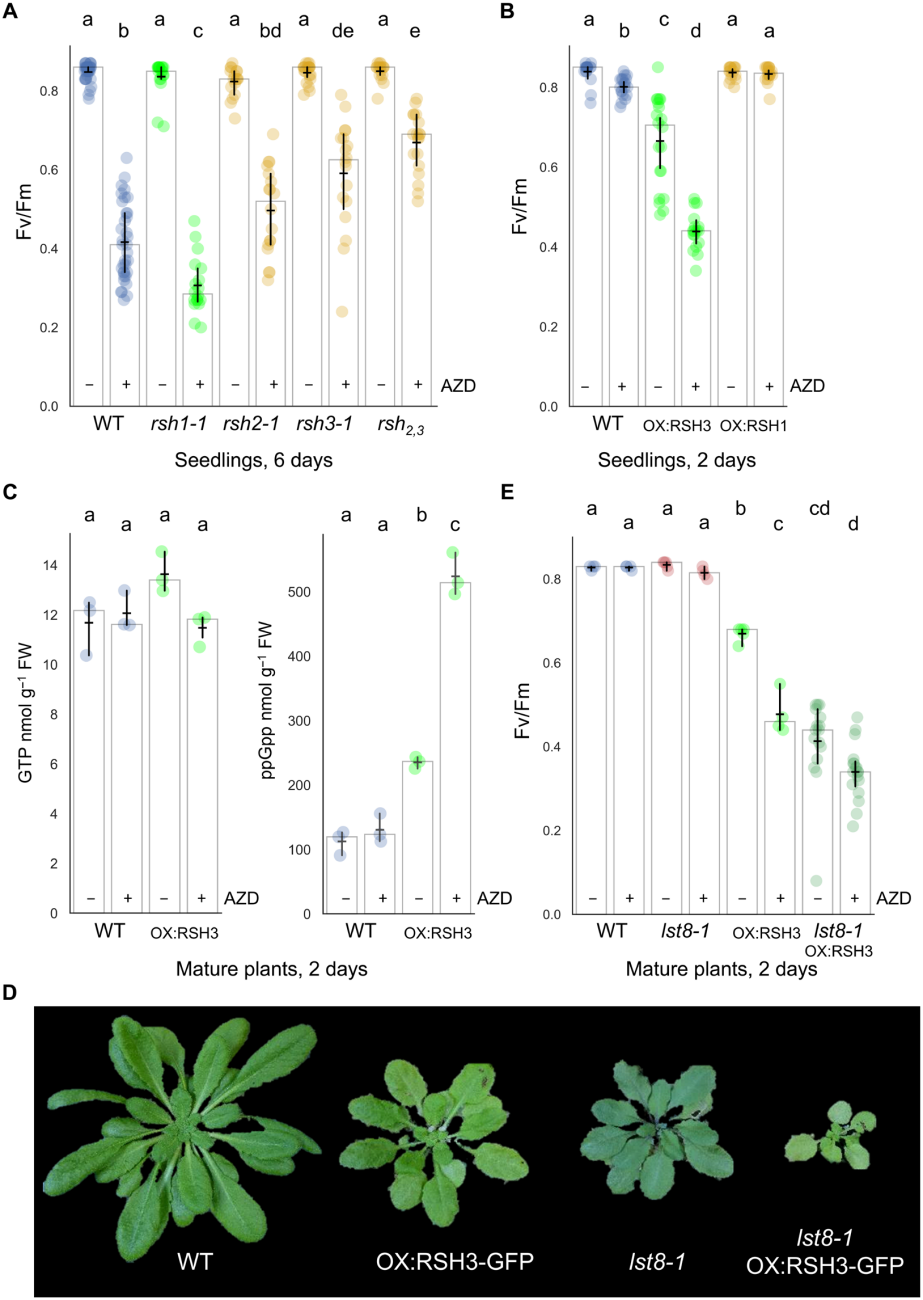
TOR activity regulates photosynthesis via RSH-dependent ppGpp synthesis. Maximal efficiency of PSII (Fv/Fm) was measured in seedlings of the indicated Arabidopsis lines treated ±10 μM AZD for (**A**) 6 days (*n* = 17 to 35 plants) and (**B**) 48 hours (*n* = 17 to 20 plants). (**C**) Nucleotide quantification in adult plants treated ± 10 μM AZD for 48 hours (*n* = 3 biological replicates). (**D**) Images of 5-week-old Arabidopsis wild-type and mutant plants grown under short-day conditions (8-hour light/16-hour dark). (**E**) Fv/Fm measurements from plants of the same age treated ±10 μM AZD for 48 hours (*n* = 4 to 20). Wild type is *qrt1-2* for (A) and Col-0 for the other panels. Graphs show mean (horizontal bar), median (column height), and 95% confidence interval (CI) (vertical line). Lowercase letters indicate statistical groups. FW, fresh weight. Source data are available in data S1.

We next sought to determine whether TOR regulates RSH-dependent ppGpp homeostasis by monitoring photosynthesis, a conserved target of ppGpp (*2*–*4*, *23*–*26*). Treatment of Arabidopsis seedlings with AZD caused a dose-dependent drop in photosynthetic efficiency. TOR-haploinsufficient seedlings were more sensitive to AZD, indicating that this effect is TOR dependent (fig. S8) (*16*). We then inhibited TOR in seedlings lacking the RSH1 ppGpp hydrolase (*rsh1-1*) or the main ppGpp synthetases RSH2 and RSH3 (*rsh2-1*, *rsh3-1*, and *rsh_2,3_*) (Fig. 2A). TOR inhibition caused a sharp drop in the maximal efficacity of photosystem II (Fv/Fm) in the wild type, and this drop was more pronounced in *rsh1-1* seedlings that lack ppGpp hydrolase activity. In contrast, we observed progressive resistance to the effect of TOR inhibition in *rsh2-1*, *rsh3-1*, and lastly *rsh_2,3_*, where the drop in Fv/Fm was strongly curtailed.

We further confirmed the effect of TOR inhibition on ppGpp homeostasis by testing plants overexpressing the RSH1 ppGpp hydrolase (OX:RSH1-GFP) and OX:RSH3-GFP plants that accumulate high ppGpp levels (*3*). OX:RSH3-GFP seedlings were hypersensitive to TOR inhibition, while OX:RSH1-GFP seedlings were resistant (Fig. 2B). OX:RSH3-GFP adult plants were also hypersensitive to TOR inhibition (fig. S9, A and B), and this was accompanied by a marked increase in ppGpp levels within 48 hours (Fig. 2C). The wild-type control did not show a significant increase in ppGpp levels in response to AZD. This correlates well with the lack of a significant drop in Fv/Fm over short treatment times in the wild type (Fig. 2B). The Fv/Fm of OX:RSH3 was also hypersensitive to nitrogen deprivation (fig. S9, C and D), a physiologically relevant stress known to inhibit TOR activity (*27*, *28*).

Together, these results point to the existence of a TOR-RSH pathway that regulates photosynthesis. However, variations in the sensitivity of *RSH* mutants and overexpressors to TOR inhibition could arise from perturbations of endogenous TOR activity by ppGpp signaling. We therefore assessed the phosphorylation status of ribosomal protein S6 (RPS6), a robust marker of TOR activity in Arabidopsis (*29*). The *RSH* mutant lines *rsh1-1* and *rsh_2,3_* exhibited similar RPS6 phosphorylation to the wild-type control whether TOR was stimulated or inhibited (fig. S10A). These results indicate that there is no substantial alteration in TOR status in the *RSH* mutants. Similarly, the RPS6 assay showed that unstimulated TOR activity in OX:RSH1-GFP and OX:RSH3-GFP lines remained comparable to wild type, suggesting no significant differences in TOR activity in the absence of stimulation (fig. S10B). However, upon stimulation, OX:RSH3-GFP lines showed attenuated RPS6 phosphorylation. This reduction in TOR stimulation might be expected given the reduced growth and photosynthetic efficiency of OX:RSH3-GFP plants under normal conditions (Fv/Fm = 0.70 for OX:RSH3-GFP versus 0.84 for wild type; Fig. 2B) and the established link between TOR activity and photosynthesis (*30*). Despite the altered stimulation, RPS6 in OX:RSH3-GFP showed a wild-type–like response to TOR inhibition, with no indication of hypersensitivity. We therefore show that ppGpp accumulation mediated by the activities of the enzymes of ppGpp metabolism (RSH2, RSH3, and RSH1) is a major driver of photosynthesis repression following the inhibition of TOR.

### Absence of LST8 exacerbates the phenotype of OX:RSH3-GFP

Next, we investigated whether loss of LST8 in the *lst8-1* mutant affected the hypersensitivity of OX:RSH3-GFP plants to AZD. Notably, *lst8-1* OX:RSH3-GFP plants showed a severe growth and development phenotype that was stronger than in the parental lines (Fig. 2D). Despite phenotypic differences, mature wild-type and *lst8-1* plants showed similar Fv/Fm ratios with or without exposure to AZD for 2 days (Fig. 2E). However, the Fv/Fm of untreated *lst8-1* OX:RSH3-GFP dropped to the same level as in AZD-treated OX:RSH3-GFP, strongly suggesting that the absence of LST8 leads to constitutive activation of RSH3 via a reduction in either TOR activity or the quantity of interaction partner (Fig. 2E). In agreement, *lst8-1* OX:RSH3-GFP appeared less sensitive to AZD treatment.

### The LST8-RSH3 interaction is required for TOR-dependent regulation of RSH3

**Fig. 3.**
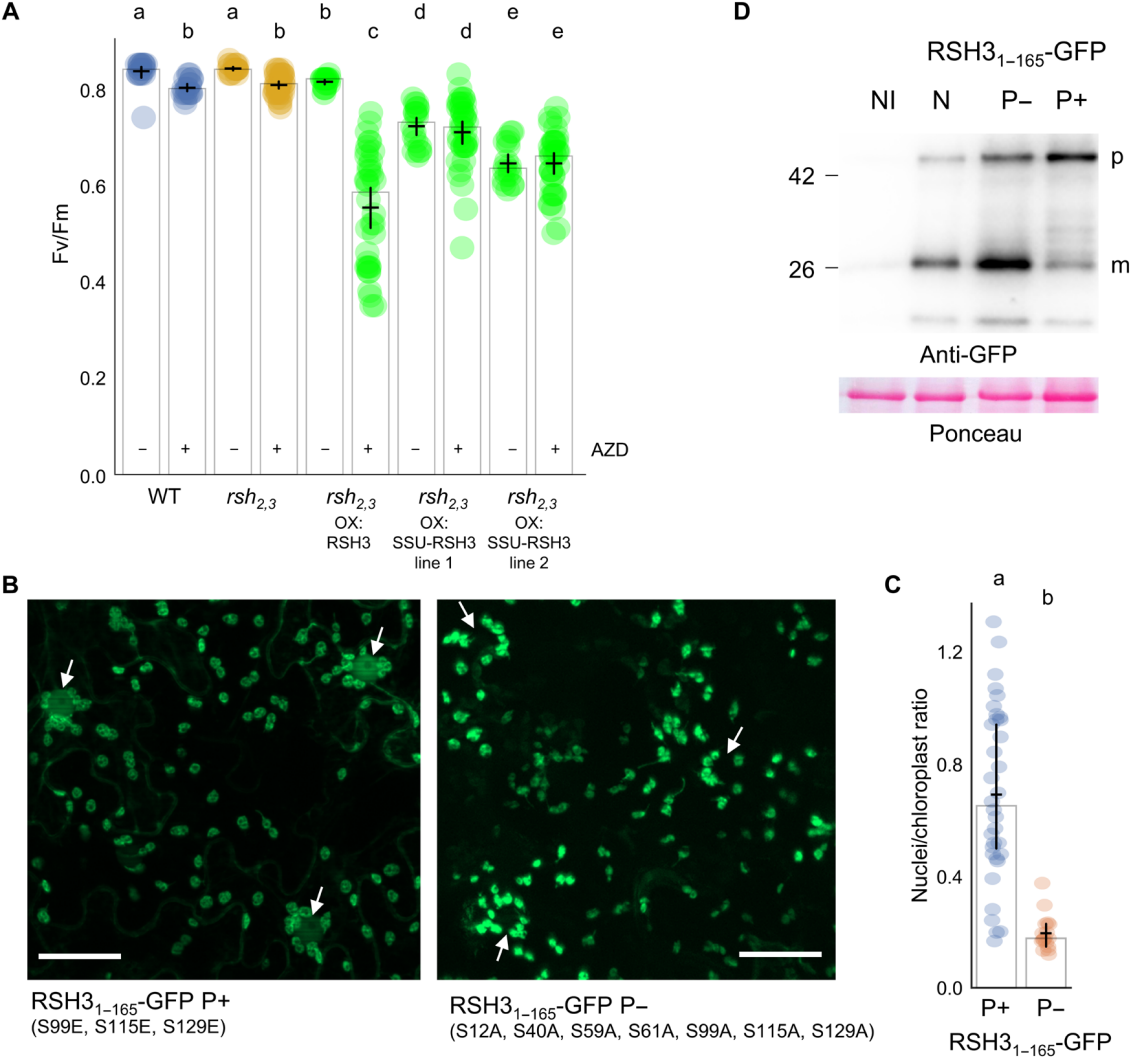
TOR regulates RSH3 activity and cellular distribution via the RSH3 NTR. (**A**) Maximal efficiency of PSII (Fv/Fm) was measured in Arabidopsis seedlings of the indicated lines treated ±10 μM AZD for 48 hours (*n* = 18 to 36 plants). RSH3, RSH3-GFP; SSU-RSH3, SSU-RSH3_65-END_-GFP; WT, wild type (*qrt1-2*). (**B**) Fluorescence microscopy images of *N. benthamiana* leaves expressing RSH3_1–165_-GFP P+ and RSH3_1–165_-GFP P−. White arrows indicate nuclei surrounded by chloroplasts. Scale bars, 50 μm. (**C**) Quantification of GFP fluorescence in nuclei against GFP fluorescence in nuclei-proximal chloroplasts, *n* = 50 nuclei. (**D**) Immunoblots of protein extracts from *N. benthamiana* expressing native (N), phosphominus (P−), or phosphomimic (P+) RSH3_1–165_-GFP and the non-inoculated control (NI). p, precursor protein; m, mature protein after chloroplast import. Graphs show mean (horizontal bar), median (column height), and 95% CI (vertical line). Lowercase letters indicate statistical groups. Source data available are in data S1.

The previous experiments lead us to ask whether the RSH3 NTR is required for the regulation of ppGpp signaling by TOR. We therefore generated a new set of plants overexpressing RSH3-GFP (OX:RSH3-GFP) or RSH3-GFP with a truncated NTR that is no longer able to interact with LST8 and the addition of the SSU CTP to assure chloroplast localization (OX:SSU-RSH3_65-END_-GFP) (Fig. 1D and figs. S3B and S4B). These lines were created in the *rsh_2,3_* background to prevent interference by the up-regulation of *RSH2/3* transcription that can occur under stress conditions (*25*). As expected, *rsh_2,3_* OX:RSH3-GFP was hypersensitive to TOR inhibition (Fig. 3A). Notably, however, *rsh_2,3_* seedlings expressing SSU-RSH3_65-END_-GFP, which cannot interact with LST8 (Fig. 1D) were completely insensitive to TOR inhibition. These results, together with the constitutive activation of RSH3 in the OX:RSH3-GFP *lst8* plants (Fig. 2E), demonstrate that the interaction between LST8 and the NTR is required for repression of RSH3 activity in planta.

We previously saw that an OX:RSH3 overexpressor showed attenuated TOR activity in response to stimulation (fig. S10B). We therefore decided to determine whether this was also the case for *rsh_2,3_* OX:RSH3-GFP plants that show a milder phenotype under standard growth conditions (Fv/Fm = 0.81) yet similar sensitivity to AZD at the level of photosynthesis (Fig. 3A). *rsh_2,3_* OX:RSH3-GFP lines did not show any variance in RPS6 phosphorylation compared to the *rsh_2,3_* control under nonstimulated, stimulated, or inhibited conditions (fig. S11). These results indicate that there is no detectable alteration in TOR activity. Furthermore, *rsh_2,3_* OX:SSU-RSH3_65-END_-GFP lines, which no longer interact with TOR, show a further reduced Fv/Fm under standard growth conditions (0.6 to 0.7; Fig. 3A) yet remain unresponsive to AZD treatment at the level of photosynthesis. Consequently, it can be inferred that the high sensitivity of photosynthesis to TOR inhibition in plants overexpressing full-length RSH3 cannot be attributed to the modification of TOR status by RSH overexpression or associated changes in ppGpp levels. This conclusion, coupled with the unchanged TOR status in *RSH* mutants (fig. S10), firmly positions the RSH-mediated regulation of photosynthesis downstream of TOR.

### NTR phosphorylation attenuates chloroplast targeting

We reasoned that phosphorylation of the RSH3 CTP might affect RSH3 activity by reducing accumulation in the chloroplast. Phosphorylation of chloroplast precursors is known to impede import (*31*). We therefore analyzed the localization of phosphomimic and phospho-null mutants of RSH3_1–165_-GFP. Phosphomimic RSH3_1–165_-GFP showed both chloroplastic and nucleocytosolic localization, while the native and phospho-null form showed only chloroplastic localization (Fig. 3C and fig. S5B). Furthermore, a greater proportion of mature protein compared to precursor was observed for the native and phospho-null RSH3_1–165_-GFP, while the phosphomimic accumulated a lower proportion of mature protein (Fig. 3D). Full-length phosphomimic RSH3-GFP also showed a reduced chloroplast localization (fig. S12). Together, these results support a model whereby TOR-dependent phosphorylation attenuates the chloroplast localization of RSH3. Inactivation of TOR, either artificially or via nitrogen starvation, leads to rapid RSH3 dephosphorylation and increased chloroplast localization to allow more ppGpp synthesis, which, in turn, inhibits photosynthesis. The inherent instability of RSH3 (fig. S4A) somewhat masks the increased level of mature RSH3 in the chloroplast (Fig. 1E). The small and transient pool of mature RSH3 might be highly sensitive to fluctuations in import of the precursor protein.

## DISCUSSION

In conclusion, we reveal a conserved posttranslational mechanism for the regulation of chloroplast function by the TOR kinase in plants. This mechanism, which is independent and distinct from transcriptional pathways of chloroplast (*7*–*9*, *32*), may allow rapid coordination of the nucleocytosolic and chloroplast compartments. Such coordination may be particularly important during episodes of stress where growth and photosynthesis must be down-regulated in lockstep to prevent photooxidative damage (*25*). Together with a recent report that TOR promotes accumulation of the chloroplast β-AMYLASE1 in stomatal chloroplasts (*32*), our work sets a precedent for the regulation of organellar function by TOR and provides a molecular mechanism for explaining the TOR-dependent regulation of photosynthesis.

## MATERIALS AND METHODS

### Plant material

Arabidopsis plant lines used and generated in this study are listed in table S2.

### Plant growth conditions

In each experiment, the seeds for each line were derived from the same batch of plants grown together. For growth on plates, seeds were surface-sterilized with 70% ethanol and 0.01% Triton X-100, rinsed with 100% ethanol, and dried and placed on square culture plates containing 45 ml of 0.5× Murashige and Skoog salts (Merck Sigma-Aldrich), 0.5 g liter^−1^ MES, and 0.8% agar (Merck Sigma-Aldrich), adjusted to pH 5.7 with KOH. Plates were placed at 4°C for 2 days in the darkness and then transferred to controlled growth conditions with 16-hour/8-hour photoperiod at 22°C day/20°C night. For nonsterile growth, *Arabidopsis thaliana* and *N. benthamiana* plants were grown in soil in a controlled environment at 120 μmol m^−2^ s^−1^ illumination with an 16-hour/8-hour photoperiod at 22°C day/20°C night (long days, unless otherwise stated) and 55% day/75% night relative humidity. Plants were treated weekly with a Coïc-Lesaint complete nutrient solution.

### Cloning

New gene parts (level 0 modules) were amplified by polymerase chain reaction (PCR) or synthesized directly (Twist Biosciences) and sequenced. The resulting modules are free from Bsa I, Bsm BI, Bpi I, and Sap I type IIS sites and can be mobilized in the MoClo cloning system (*33*). Restriction ligation reactions for the assembly of transcriptional units (level 1) and assemblies of transcriptional units (level 2) were performed using a single step protocol as described previously with small modifications (*34*) and according to the MoClo Golden Gate assembly standard (*35*, *36*) [for detailed instructions, see the cloning guide of Velay *et al.* (*37*)]. Briefly, 100 fmol of each insert plasmid and 50 fmol of acceptor plasmid were mixed with restriction enzyme (Bpi I or Bsa I) and T4 DNA ligase in restriction enzyme buffer and 1 mM ATP in 20 μl reactions and incubated at 37°C for 5 hours. A 1.5-μl aliquot was transformed into DH10B *Escherichia coli* cells by electroporation and transformants selected on appropriate antibiotics. Correct assembly was confirmed by digestion. A list of genetic constructions can be found in table S3, and full sequences are available in the supplementary data files (data S1). All other level 0 and infrastructure modules used were described previously (*35*–*37*). The RSH3 phosphodefective peptide was constructed by mutating all serine residues that we initially predicted to be susceptible to TOR-dependent regulation, as well as S61 whose phosphorylation was experimentally identified (see also fig. S6) (*38*). The RSH3 phosphomimic peptide was constructed by mutating serine residues after the predicted CTP cleavage site at position 64.

### Y2H screening

Y2H screening was performed by Hybrigenics Services (Paris, France). The coding sequence of LST8-1 (At3g18140, residues 1 to 305) was amplified from Arabidopsis cDNA and cloned into pB66 (GAL4 N-terminal fusion) as baits. The prey library was derived from 1-week-old Arabidopsis seedlings. In total, 69 million (pB66_C) interactions were examined, and 89 clones were processed. Four clones containing fragments of RSH2 were identified with high confidence in the interaction, and two clones containing fragments of RSH3 were identified with good confidence in the interaction.

### Transient expression by agroinfiltration

*Agrobacterium tumefaciens* GV3101 transformed with plant expression constructs were grown at 28°C overnight in LB medium supplemented with rifampicin and a selective antibiotic. The cultures were then diluted to an optical density at 600 nm of 0.2 in infiltration buffer containing 10 mM MES (pH 5.5), 10 mM MgCl_2_, and 200 μM acetosyringone and then infiltrated into leaves of 1-month-old *N. benthamiana* plants using a 1-ml syringe. Infiltrated plants were returned to standard growth conditions for 3 days before observation or further treatment.

### Biotin labeling

Leaf discs or whole leaves were taken from *N. benthamiana* plants 3 days after agroinfiltration and vacuum-infiltrated with a solution of 50 μM biotin. The plant material was then floated on water and transferred to standard growth conditions for 30 min or 2 hours before being rinsed with cold water and flash-frozen in liquid nitrogen. Total proteins were extracted in SDS sample buffer and analyzed directly by SDS–polyacrylamide gel electrophoresis (SDS-PAGE) or further processed to isolate biotinylated proteins or for immunoprecipitation.

For the isolation of biotinylated proteins, the protein extract was precipitated in four volumes of acetone and washed twice with 400 μl of acetone to remove free biotin. The proteins were then resuspended in binding buffer [100 mM tris-HCl (pH 7.5), 2% SDS, and 8 M urea] and incubated with 150-μl streptavidin magnetic beads (New England Biolabs) for 3 hours with agitation. Beads were washed twice in binding buffer, twice with 1 M NaCl in 100 mM tris-HCl (pH 7.5), once with double distilled water, and once with ammonium bicarbonate (pH 8.0). Success of the enrichment procedure was confirmed by analyzing 5% of beads by SDS-PAGE and immunoblotting. Enriched proteins were then identified by LC–tandem mass spectrometry (MS/MS).

### Immunoblotting and protein detection

Total leaf proteins were extracted in SDS sample buffer and separated by SDS-PAGE as described previously (*25*). Proteins were transferred onto a nitrocellulose membrane and probed with specific antibodies. The following primary antibodies were used against the HA tag (monoclonal ab9110, Abcam; 1/5000), GFP/CFP (polyclonal A-11122, Thermo Fisher Scientific; 1/5000), RPS6 (Cell Signaling, #2317; 1/1000), P-RPS6 (1/5000) (*29*), and PBA1 (Abcam, ab98861; 1/2000). Biotinylated proteins were detected directly using streptavidin–horse radish peroxidase conjugate (RPN1231, Cytiva). Total proteins were visualized after separation and transfer using Ponceau Red protein stain. Original gel images are provided in the supplementary data files (data S1).

### Immunoprecipitation

Total leaf proteins were extracted from 150-mg *N. benthamiana* powdered leaf disks in 1 ml of extraction buffer [25 mM tris-HCl (pH 7.5), 150 mM NaCl, 2 mM EDTA, 1% Triton X-100, 0.1% SDS, cOmplete protease inhibitor cocktail (Roche), phosphatase inhibitor cocktail 2 (Sigma-Aldrich), and phosphatase inhibitor cocktail 3 (Sigma-Aldrich)]. Twenty-five microliters of α-GFP nanobody:Halo:His6 coupled to Magne HaloTag beads (Promega) was added to each sample, which were incubated 2 hours at 4°C on a rotating wheel. After incubation, beads were magnetically separated and then washed twice with extraction buffer and once with double distilled water. Proteins were then either analyzed by SDS-PAGE after eluting from the beads by heating with SDS sample buffer at 95°C for 10 min or analyzed by LC-MS/MS. The α-GFP nanobody:Halo:His6 used for immunoprecipitation was produced using an α-GFP-nanobody:Halo:His6 construct (Addgene plasmid #111090) and prepared as described by Chen *et al*. (*39*).

### Identification of peptides by LC-MS

Proteins enriched on streptavidin or α-GFP nanobody:Halo:His6 beads were submitted to on-bead digestion or were eluted and subsequently submitted to a S-trap digestion according to the manufacturer’s procedure (ProtiFi). For on-bead digestion, the beads were washed once with 160 μl of 100 mM NH_4_HCO_3_,/CH3CN (v/v, 1/1), then reduced with 50 μl of 10 mM dithiothreitol in 100 mM NH_4_HCO_3_ for 45 min at 25°C in the dark, and alkylated with 50 μl of 55 mM iodoactetamide in 100 mM NH_4_HCO_3_ for 30 min at 25°C in the dark. The beads were washed once again as above and then resuspended in 50 μl of 25 mM NH_4_HCO_3_ with 0.025% ProteaseMAX (v/v) and 150 ng of trypsin/LysC. The digestion was continued overnight at 37°C with agitation at 500 rpm. The free digested peptides were collected in a clean tube, and the beads were washed twice with 25 μl of 0.1% in water (agitation, 5 min; 500 rpm) and 25 μl of CH3CN (agitation, 5 min; 500 rpm). All supernatants were pooled, dried down, resuspended in 0.05% trifluoroacetic acid/2% acetonitrile in water, and quantified by the quantitative colorimetric peptide assay (Thermo Fisher Scientific). Peptides (600 ng) were injected on a reversed phase C18 column (Acclaim PepMap RSLC, 75 μm by 150 mm, 2 μm) and separated on a two-step linear gradient from 6 to 40% in 52 min of mobile phase B [80% acetonitrile/0.1% formic acid (FA) in water (v/v)] in mobile phase A [0.1% FA in water (v/v)] and then from 40 to 65% of B in A for 11 min, followed by a 5-min chase at 99% of B. After ionization in the nanosource (source Easy Spray, Thermo Fisher Scientific) at 1.9-kV spray voltage (capillary temperature set at 275°C), the peptides were detected into the mass spectrometer Q-Exactive plus (Thermo Fisher Scientific) in positive ion mode. A top 10 data-dependent acquisition mode was applied, alternating a scan event full MS in the Orbitrap analyzer at 70,000 resolution in a 350 to 1900 mass/charge ratio range and scan events of fragmentation (MS/MS) of the 10 top ion parents, in the higher-energy collisional dissociation cell, at 17,500 resolution, with a dynamic exclusion of 30 s.

Spectra were processed in Proteome Discoverer (Thermo Fisher Scientific, version: 2.4.1.15) using the algorithm Sequest HT and a peptide validator node based on Peptide Spectral Match level with a maximum Delta Cn of 0.05. LC-MS data were searched against an *N. benthamiana* protein database (*40*), target protein sequences (TID-LST8, TID-YFP, RSH3-GFP, and RSH3_39–221_-GFP), and a list of common contaminants. The protease was set as trypsin with up to three missed cleavages possible. Static modifications included carbamidomethylation (+57.021 Da), and dynamic modifications included acetyl of the N terminus (+42.011 Da), methionine oxidation (+15.995 Da), Met-loss (−131.040 Da), Met-loss + acetyl (−89.030 Da), lysine biotinylation (+226.78 Da) in the sample containing TID, and serine or threonine phosphorylation (+79.966 Da). A maximum of four dynamic modifications per peptide was allowed. Phosphorylation sites on peptides were considered only at rank 1. Peptides identifying proteins were validated with the best peptide-spectrum match (PSM) score calculated on PSM level false discovery rate (FDR) (0.01 < target FDR < 0.05). Phosphopeptide identification and summaries are available in the supplementary data files (data S1), and the raw LC-MS data files are available at the ProteomeXchange Consortium (http://proteomecentral.proteomexchange.org) via the PRoteomics IDEntifications (PRIDE) partner repository with the dataset identifier PXD047124 (*41*).

### Detection of phosphoforms by phos-tag

Total leaf proteins were extracted in SDS sample buffer as described previously (*25*). EDTA present in the sample buffer was quenched with 10 mM MnCl_2_ before protein denaturation and loading in the gel. Phos-tag gels were obtained by adding 25 μM phos-tag (Origin) and 50 μM MnCl_2_ to the 9% acrylamide/bis-acrylamide SDS-PAGE preparation. Protein resolution was carried at 20 mA per gel, and after migration, MnCl_2_ present in Phos-tag gels was quenched by a 30-min wash in 10 mM EDTA. Proteins were transferred onto a nitrocellulose membrane and probed with primary antibodies against GFP. Phos-tag gels were compared with standard SDS-PAGE to identify phosphorylated proteins.

### TOR inhibition treatments

We used three different TOR inhibition protocols depending on the type and growth stage of the plant. A stock solution of 10 mM AZD-8055 (Tocris) in dimethyl sulfoxide (DMSO) was used for preparing buffers and media for TOR inhibition (10 μM final concentration in all cases, except for the RPS6 experiments where 2 μM was used). For the 0 μM AZD-8055 control, an equivalent quantity of DMSO was used.

For the inhibition of TOR in *N. benthamiana*, 6-mm leaf disks were removed from agroinfiltrated leaves and vacuum-infiltrated with liquid half-strength MS solution containing 10 μM AZD-8055. Then, leaf disks were floated on 10 ml of the respective treatment solution in round culture dishes under standard growth conditions for 2 hours. After incubation, discs were frozen, and total proteins were extracted as described below.

For the inhibition of TOR in Arabidopsis seedlings, developmentally homogeneous 5-day-old Arabidopsis seedlings of each genotype were transferred onto new square culture plates containing 45 ml of growth medium in a 6-by-3 grid pattern. Seedlings were treated by placing 45 1-μl drops of 10 mM AZD-8055 stock solution (or the same volume of DMSO for the control) equally spaced between the seedlings for a final concentration of 10 μM AZD-8055 in the plate.

For the inhibition of TOR in mature Arabidopsis plants, entire rosettes were treated by spraying with liquid half-strength MS solution containing 10 μM AZD-8055 or the equivalent quantity of DMSO. The rosettes were sprayed twice at 24-hour intervals, and plants were analyzed 48 hours after the first treatment.

### Nitrogen limitation treatment

For nitrogen limitation, plants were germinated on nitrogen replete half-strength MS solution. After 5 days, seedlings were transferred to square culture plates containing nitrogen replete (+N) [0.5× Murashige and Skoog salts (Caisson Labs), 1% sucrose, MES (0.5 g liter^−1^), and 0.4% phytagel (Merck Sigma-Aldrich), adjusted to pH 5.7 with KOH] or nitrogen limiting (−N) [+N medium diluted 1/25 in 0.5× Murashige and Skoog medium without nitrogen (Caisson Labs), MES (0.5 g liter−1), and 0.4% phytagel (Merck Sigma-Aldrich), adjusted to pH 5.7 with KOH] growth medium. Plants were then returned to standard growth conditions.

### RPS6 phosphorylation assay

Growth conditions and media for the RPS6 phosphorylation assay were as previously described (*28*, *42*). Briefly, seeds were germinated on vertical plates containing complete medium with reduced concentrations of KNO_3_ (1 mM) and sucrose (0.3%). Five-day-old seedlings were then transferred to complete liquid media without sucrose (−Suc). After 24 hours, the medium was replaced with fresh medium (−Suc), medium supplemented with 1% sucrose (+Suc, stimulation), or medium supplemented with 1% sucrose and 2 μM AZD-8055 (+Suc + AZD, inhibition) and incubated for 5 hours. Seedlings from each of the three treatments were snap-frozen in liquid nitrogen, and proteins were extracted in SDS sample buffer supplemented with 1% phosphatase inhibitor cocktail 3 (Merck Sigma-Aldrich). After immunoblotting as described above, the phospho-RPS6 to RPS6 ratio was determined by quantification of bands by densitometry.

### Microscopy

Leaf discs were analyzed 3 days after agroinfiltration. The discs were mounted in perfluorodecalin (Merck Sigma-Aldrich) as described previously (*43*). Capture of fluorescence images was performed using the AxioImager APO Z1 microscope (Zeiss) using the following filters: chlorophyll: excitation, 625 to 655 nm; emission, 665 to 715 nm; mCherry: excitation, 533 to 558 nm; emission, 570 to 640 nm; GFP/GFP: excitation, 455 to 495 nm; emission, 505 to 555 nm; YFP: excitation, 455 to 495 nm; emission, 515 to 555 nm; and CFP: excitation, 431 to 441 nm; emission, 460 to 500 nm. Standard exposure times of 10 ms for chlorophyll and 50 to 200 ms for fluorescent proteins were kept for all observations. No fluorescence bleed-through was observed between the different fluorescent protein channels. Images were captured from different regions of each inoculated leaf and from at least two leaves per experiment. Ten-micrometer-deep Z stacks composed of 21 slices were acquired and then converted into maximum intensity projections in the ZEISS Efficient Navigation (ZEN) software (Zeiss). Postacquisition image processing was then performed using ImageJ (*44*, *45*). Unprocessed images were used for the quantification of normalized fluorescence intensities. The integrated signal density was calculated for the enhanced GFP channel. Fluorescence localized in nuclei was divided by the average fluorescence present in the nuclei-attached chloroplast.

### Chlorophyll fluorescence measurements

Plants were dark-adapted for 20 min, and chlorophyll fluorescence was measured in a Fluorcam FC 800-O imaging fluorometer (Photon System Instruments). PSII maximum quantum yield (Fv/Fm) was calculated as (Fm − Fo)/Fm. All experiments on photosynthetic parameters were repeated independently two to five times with similar results.

### Nucleotide quantification

Nucleotides were extracted from about 150 mg of plant material and quantified by high-performance liquid chromatography–MS/MS using stable isotope-labeled ppGpp and GTP standards as described previously (*46*).

### Phylogenetic inference

Multiple-sequence alignments of homologous proteins were performed using MAFFT v7.40262 with option –auto (*47*). Alignments were then trimmed to include only the NTR up to the start of the (p)ppGpp hydrolase domain. Phylogenetic reconstructions were created using maximum likelihood with the IQ-TREE web server version 1.6.1164 using default settings, with LG + F + R7 automatically selected as the best fit evolutionary model based on Bayesian information criterion (BIC) values by ModelFinder (*48*). Branch support was tested using two methods: ultrafast bootstrap approximation using 1000 bootstraps and the nonparametric Shimodaira-Hasegawa–like approximate likelihood-ratio test. The alignments and trees are available in the supplementary data files (data S1).

### Creation of *rsh_2,3_* complementation lines

RSH3_65–715_ was amplified by PCR and assembled with the RSH3_1–64_ region in a Bsa I MoClo reaction to make a 35S:RSH3_1–715_-GFP-35S terminator level 1 module. RSH3_65–715_ was also assembled with the CTP of the Rubisco small subunit 1A NT2 module in a Bsa I MoClo reaction to make a 35S:SSU-RSH3_65–715_-GFP-35S terminator level 1 module. The level 1 modules were assembled with the OLE1:RFP reporter module (*35*, *49*) in a Bpi I MoClo reaction to make level 2 multigenic constructs. The resulting constructs were transferred into *A. tumefaciens* (strain GV3101) and used to transform *rsh_2,3_* plants by floral dipping. The majority of the recovered lines were silenced or showed very low expression. The following nonsilenced lines were obtained: two *rsh_2,3_* OX:RSH3-GFP lines, four *rsh_2,3_* OX:SSU-RSH3-GFP lines, and one *rsh_2,3_* P- OXRSH3-GFP line.

### Data analysis

Graphs and statistical tests were generated in Python (Python Software Foundation, https://python.org/) using the Panda (*50*), Matplotlib (*51*), and Seaborn (*52*) libraries. Statistical tests were performed using the Pingouin (*53*) library. A Games-Howell post hoc test was adopted for nonparametric data comparisons and a pairwise *t* test using the Benjamini-Hochberg FDR correction for multiple comparisons of data with normal distributions. Scripts and source data are available in the supplementary data files (data S1).

## Supplementary Materials

### This PDF file includes:

Figs. S1 to S12 Tables S1 to S3 Data S1 References
