# Supplementary material for: Posttranslational regulation of photosynthetic activity via the TOR kinase in plants
Source: Sci Adv. 2024 Jun 19;10(25):eadj3268. doi: 10.1126/sciadv.adj3268 (PMC11186500; doi:10.1126/sciadv.adj3268)
Supplement: Supplementary file 1 — Figs. S1 to S12 Tables S1 to S3 Data S1 References [file sciadv.adj3268_sm.pdf]

Science Advances
AAAS

Supplementary Materials for

Posttranslational regulation of photosynthetic activity via the TOR kinase in plants

Stefano D'Alessandro *et al.*

Corresponding author: Ben Field, ben.field@univ-amu.fr; Stefano D'Alessandro, stefano.dalessandro@unito.it

Sci. Adv. **10**, eadj3268 (2024)
DOI: 10.1126/sciadv.adj3268

This PDF file includes:

Figs. S1 to S12
Tables S1 to S3
Data S1
References

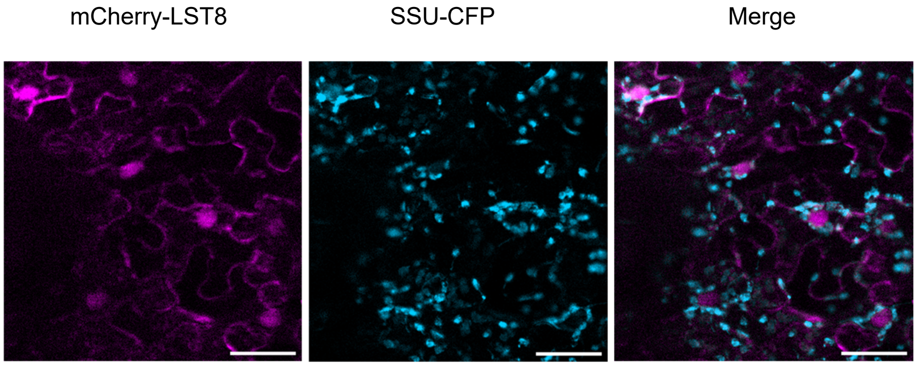

Fig. S1.

LST8 shows a nucleocytosolic localisation. Fluorescence microscopy images of *N. benthamiana* leaves expressing mCherry-LST8 and the chloroplast marker SSU-CFP. Scale bar, 50 µm.

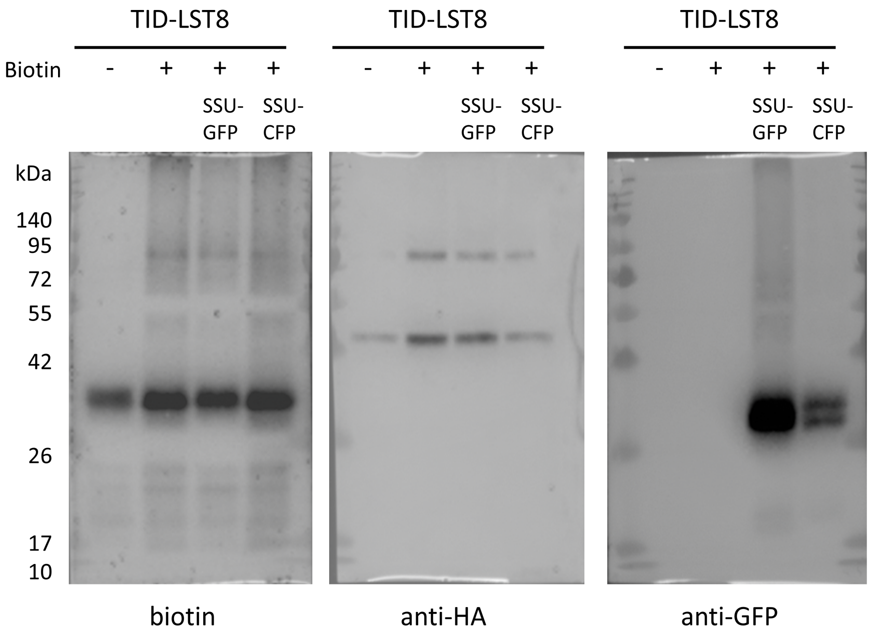

Fig. S2.

TID-LST8 proximity labelling against SSU-CFP and SSU-GFP. Blots of protein extracts from *N. benthamiana* co-expressing TID-LST8 and SSU-CFP or SSU-GFP. Leaves were infiltrated with buffer supplemented (+) or not (-) with 50 uM biotin for 2 hrs before protein extraction. Western blot analysis was conducted using anti-biotin (SBP-HRP), anti-HA (TID-LST8), and anti-GFP antibodies. Source data available in Data S1.

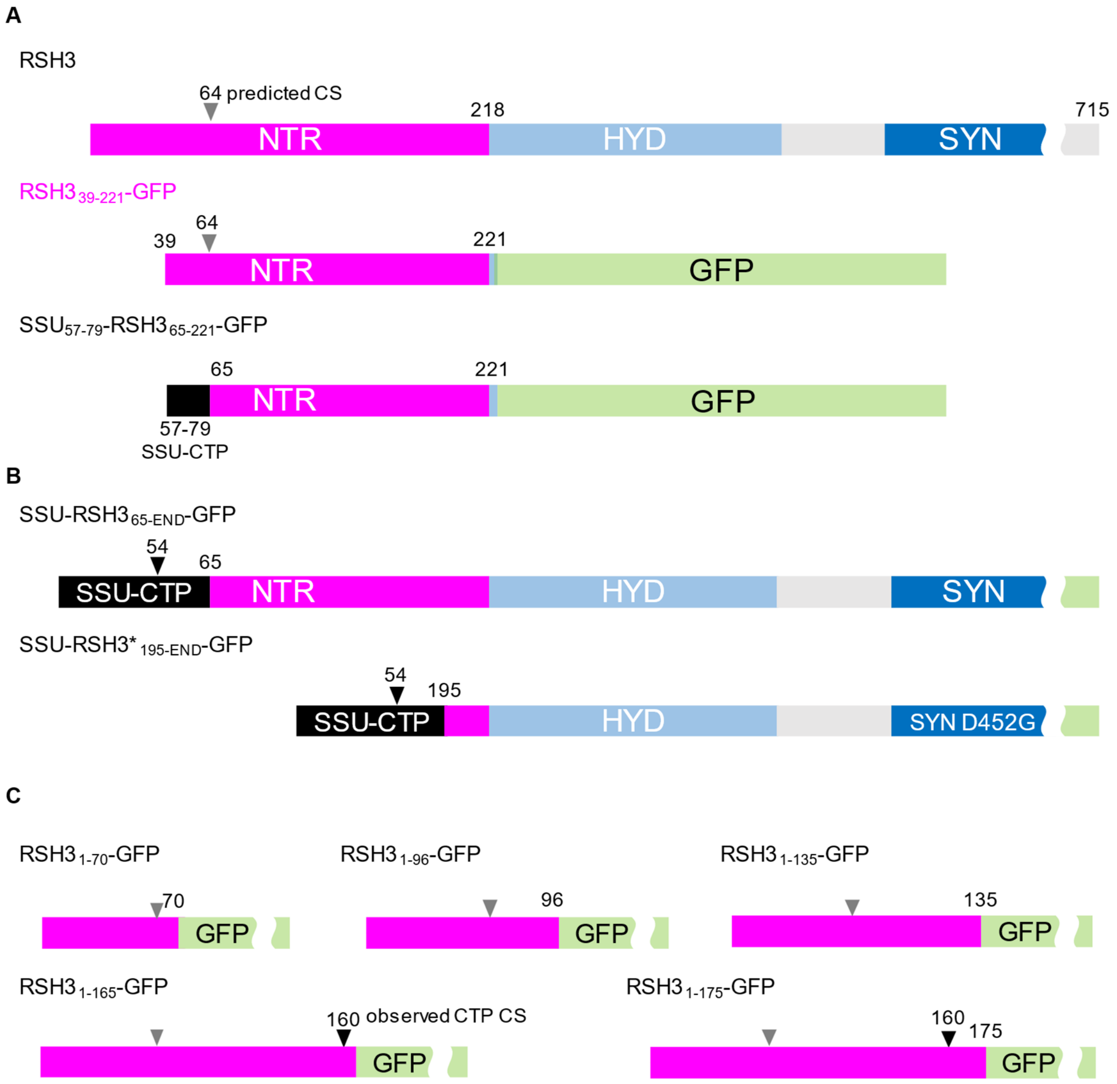

Fig. S3.

Outline of different RSH3 fusion proteins. (A) RSH3 fusions used for analyzing RSH3 control by TOR in *N. benthamiana*. (B) RSH3 with long or truncated N-terminal region targeted to chloroplast using the Rubisco small subunit CTP. Asterisk (*) indicates an inactivating mutation in the RSH3 synthetase domain (SYN D452G). (C) Series of RSH3 NTR truncations fused to GFP for determining the minimal sequence required for chloroplast targeting. Chloroplast targeting peptide (CTP), N'-terminal region (NTR, magenta), ppGpp hydrolase domain (HYD, light blue), synthetase domain (SYN, blue). TargetP predicted chloroplast import cleavage site (CS) indicated by grey arrows, and observed cleavage sites indicated by black arrows.

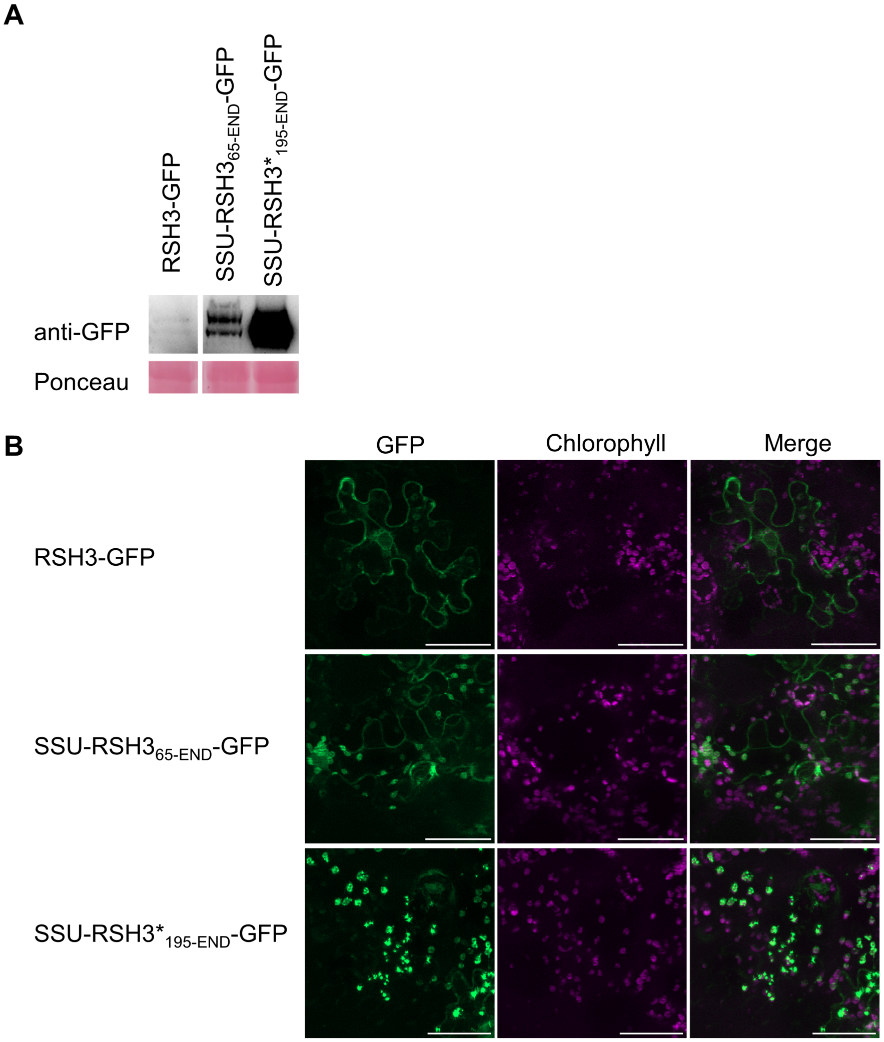

Fig. S4.

The RSH3 NTR restricts RSH3 accumulation. (A) Immunoblots of protein extracts from *N. benthamiana* expressing RSH3-GFP, SSU-RSH3$_{65\text{-END}}$-GFP and SSU-RSH3*$_{195\text{-END}}$-GFP. (B) Fluorescence microscopy images of *N. benthamiana* leaves expressing the same proteins. Scale bar, 50 µm. Source data available in Data S1.

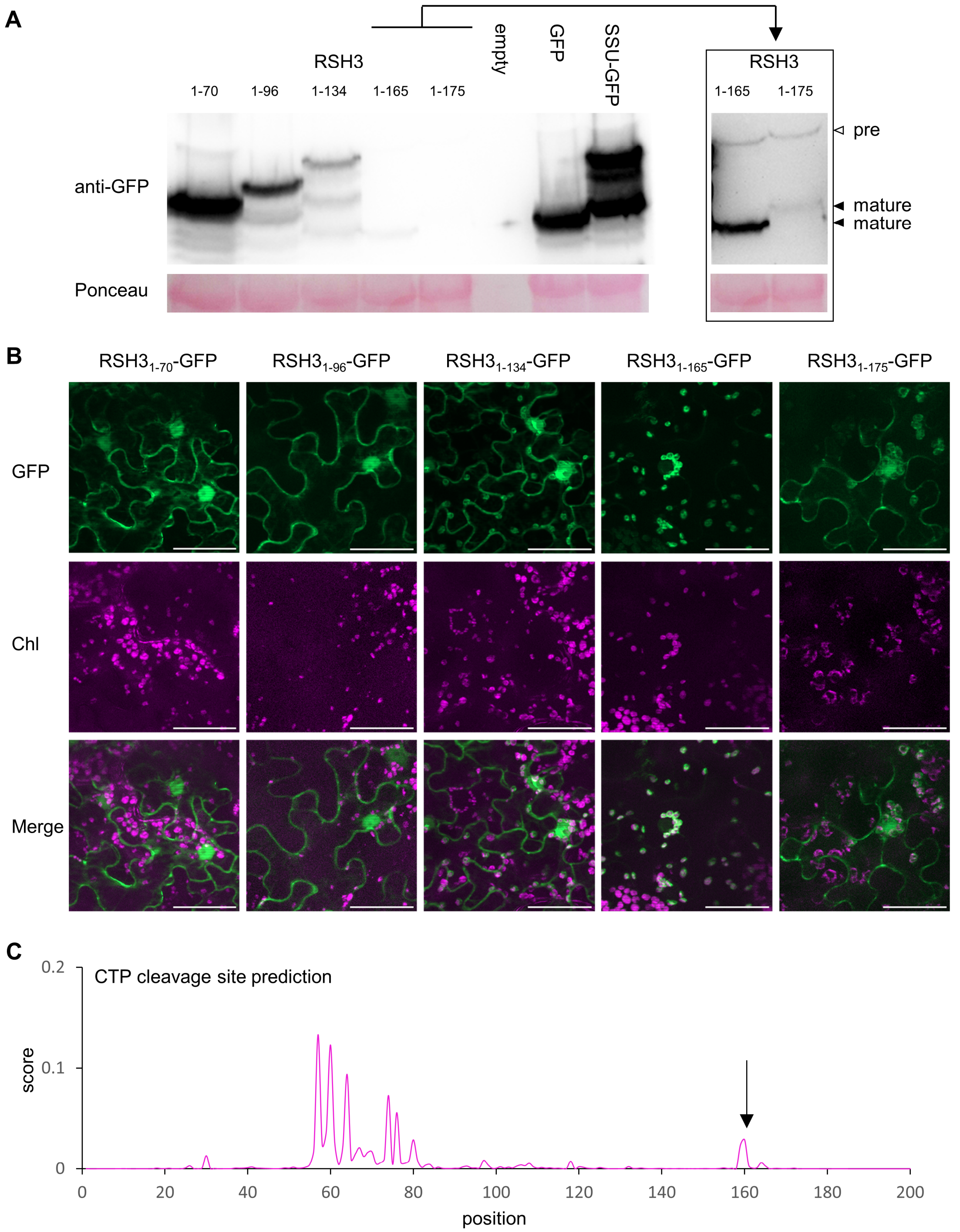

Fig. S5.

The RSH3 NTR contains an unusually long chloroplast transit peptide. A series of RSH3 N-terminal regions of increasing size were expressed in *N. benthamiana* and analyzed by (A) immunoblotting protein extracts and (B) fluorescence microscopy. Scale bar, 50 µm. (C) TargetP (*54*) prediction of RSH3 chloroplast cleavage sites (bottom). Source data available in Data S1.

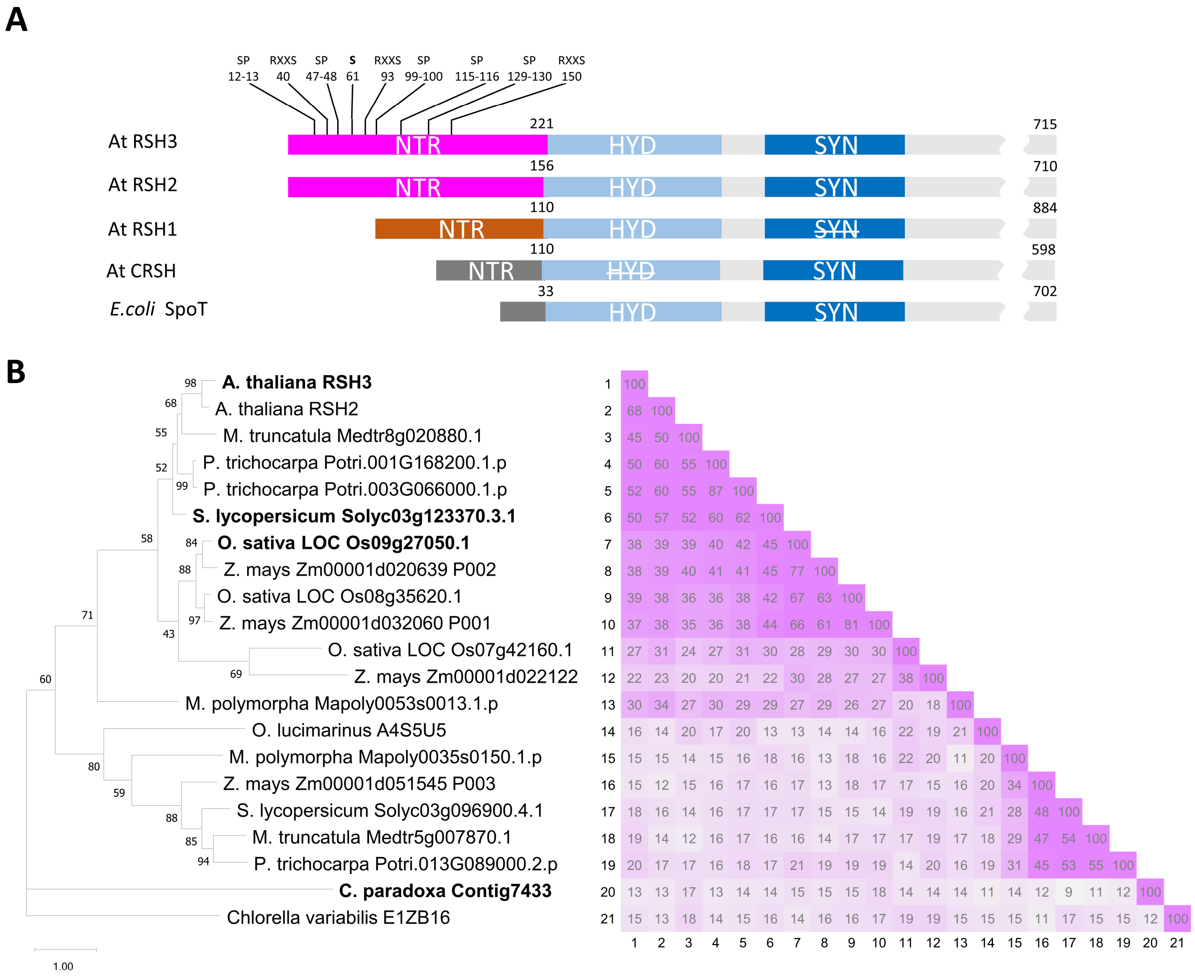

Fig. S6.

Features and evolution of the RSH NTR. (A) Domain structure of the four Arabidopsis RSH enzymes focused on the N-terminal and catalytic domains. Domains with the same colour show strong sequence homology. Serines in TOR-dependent phosphorylation contexts (SP, RXXS) are indicated above RSH3 (*19*). Prior experimental evidence existed for S61 phosphorylation (PhosPhAt 4.0)(*38*). (B) Maximum likelihood inference of evolutionary relationship between NTRs from RSH2/3 family enzymes found in plants, green algae and the glaucophyte *Cyanophora paradoxa*. NTRs tested for TOR-dependent phosphorylation are in bold. Boostrap support shown at branch nodes, and scale indicates substitutions per site. Source data available in Data S1.

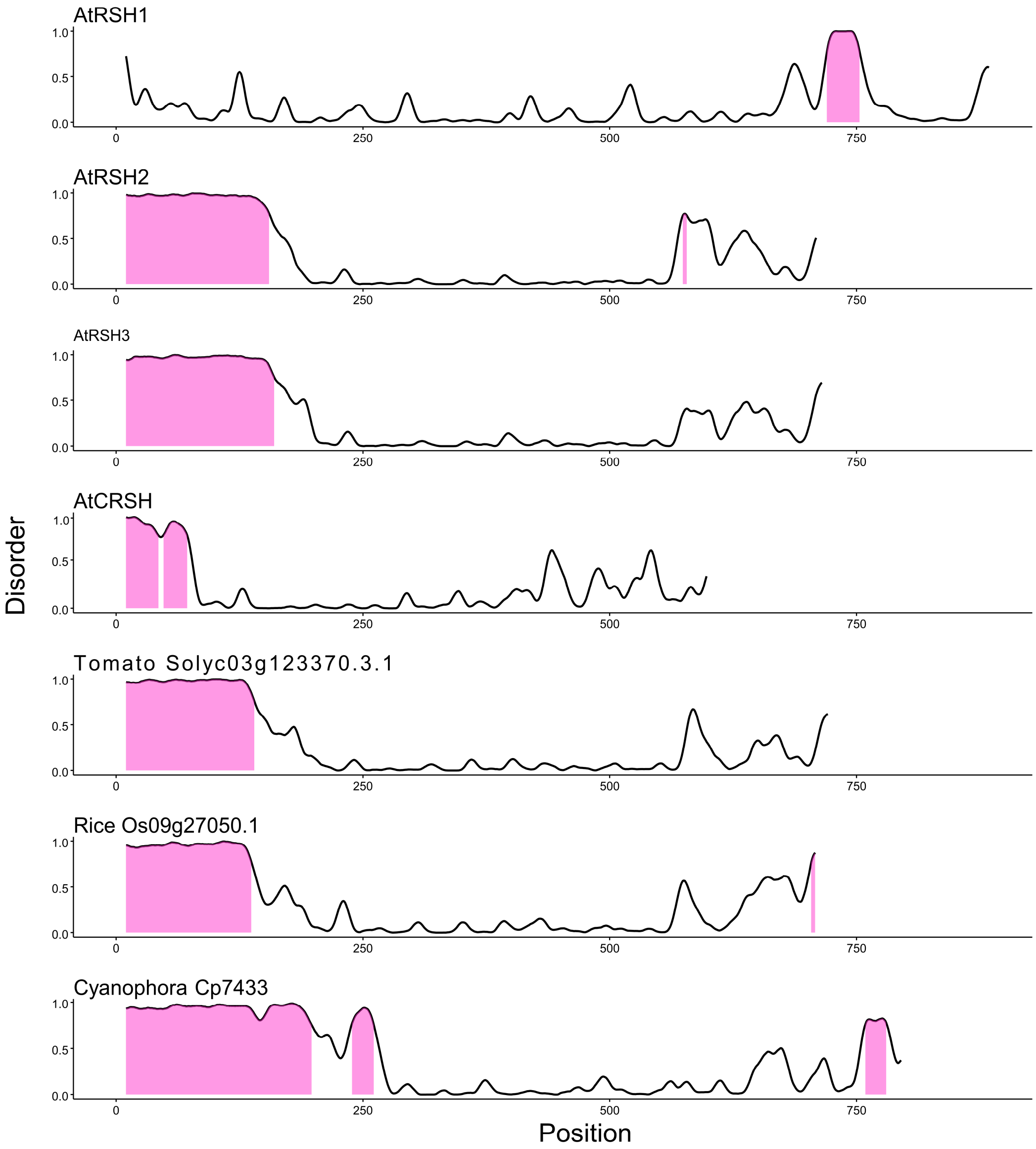

Fig. S7.

Disordered regions in RSH enzymes. Disorder prediction (10 point moving average) across Arabidopsis RSH enzymes from the RSH1, RSH2/3 and RSH4/CRSH families. Also shown are predictions for RSH2/3 family enzymes from tomato, rice and *Cyanophora paradoxa*. Regions where predicted disorder is greater than 0.75 are colored. Predicted using Metapredict v2.4 (*55*, *56*). Source data available in Data S1.

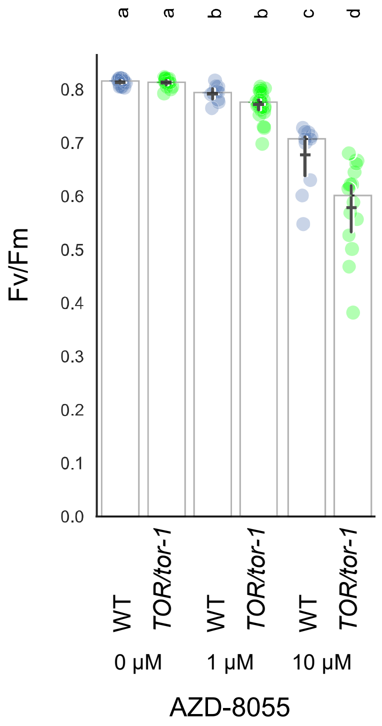

Fig. S8.

TOR-inhibition by AZD-8055 leads to a dose dependent decrease in Fv/Fm. Maximal efficiency of PSII (Fv/Fm) was measured in seedlings of wild type (Wassilewskija ecotype) and TOR/tor-1 heterozygous plants after 6 days growth on the indicated concentrations of AZD-8055. Please note that the Wassilewskija ecotype has a lower sensitivity to AZD-8055 than Col-0 which is used in the majority of other experiments (*16*). Graphs show mean (horizontal bar), median (column height) and 95% CI (vertical line). Lower-case letters indicate statistical groups. Source data available in Data S1.

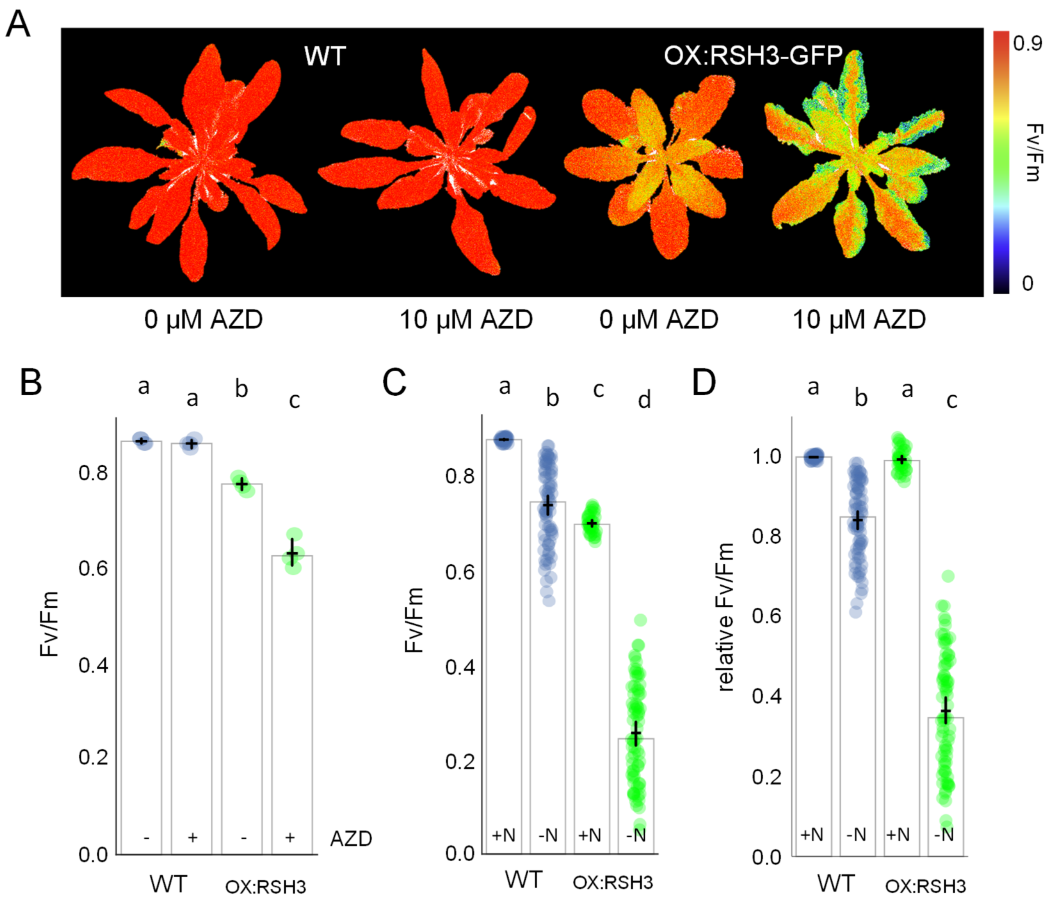

Fig. S9.

OX:RSH3 plants are hypersensitive to TOR inhibition and nitrogen starvation. Maximal efficiency of PSII (Fv/Fm) was (A) imaged and (B) quantified in 5-week-old wild type and OX:RSH3-GFP (OX:RSH3) plants treated with ± 10 µM AZD for 48 hours (n= 4 plants). Fv/Fm was measured in wild type and OX:RSH3-GFP (OX:RSH3) seedlings following transfer to a nitrogen-replete (+N) or nitrogen-limited medium (-N) for 16 days and is presented as either unmodified Fv/Fm (C), or as relative Fv/Fm (D) where values were normalized to the corresponding +N control (+N, n=36 plants; -N, n=72 plants). Graphs show mean (horizontal bar), median (column height) and 95% CI (vertical line). Lower-case letters indicate statistical groups. Source data available in Data S1.

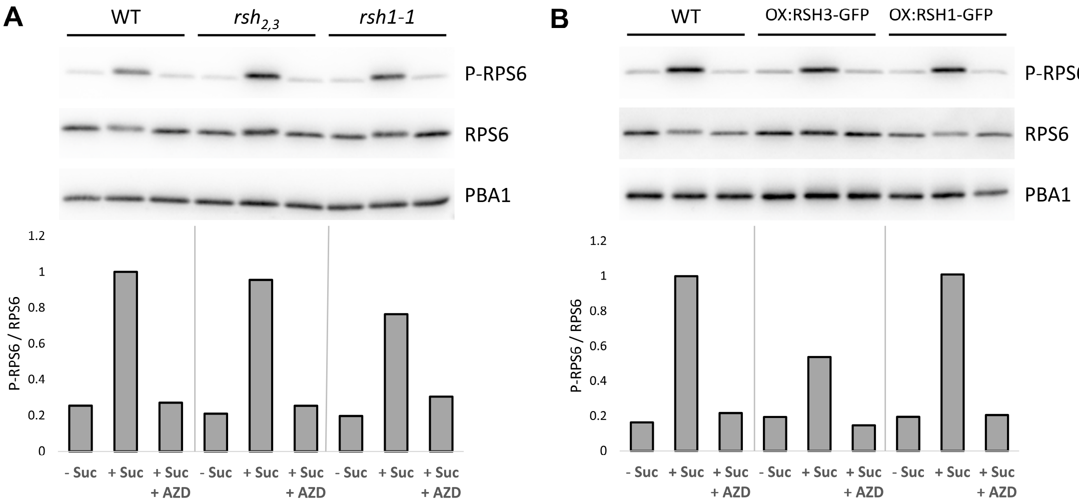

Fig. S10

RPS6 TOR activity assay on *RSH* mutants and overexpressors. Five-day-old seedlings were transferred to liquid media without sucrose and then treated for 5 hours in media with or without 1% sucrose (±Suc), or with sucrose and 2 µM AZD-8055 (+Suc +AZD). Immunoblots with the indicated antibodies were performed after SDS PAGE separation of 10 µg total proteins from (A) *RSH* mutant lines and (B) *RSH* overexpressor lines and the corresponding wild-type controls (WT). The results represent the average of two independent experiments. RPS6, total RPS6; P-RPS6, Ser240-phosphorylated RPS6; PBA1, 20S proteasome subunit PBA1. Source data available in Data S1.

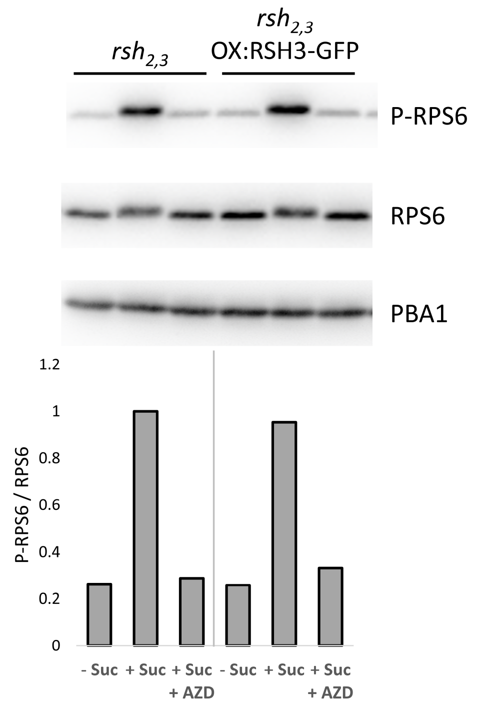

Fig. S11.

RPS6 TOR activity assay on *rsh*$_{2,3}$ OX:RSH3-GFP. Five-day-old seedlings were transferred to liquid media without sucrose and then treated for 5 hours in media with or without 1% sucrose (±Suc), or with sucrose and 2 µM AZD-8055 (+Suc +AZD). Immunoblots with the indicated antibodies were performed after SDS PAGE separation of 10 µg total proteins from *rsh*$_{2,3}$ and *rsh*$_{2,3}$ OX:RSH3-GFP. The results represent the average of two independent experiments. RPS6, total RPS6; P-RPS6, Ser240-phosphorylated RPS6; PBA1, 20S proteasome subunit PBA1. Source data available in Data S1.

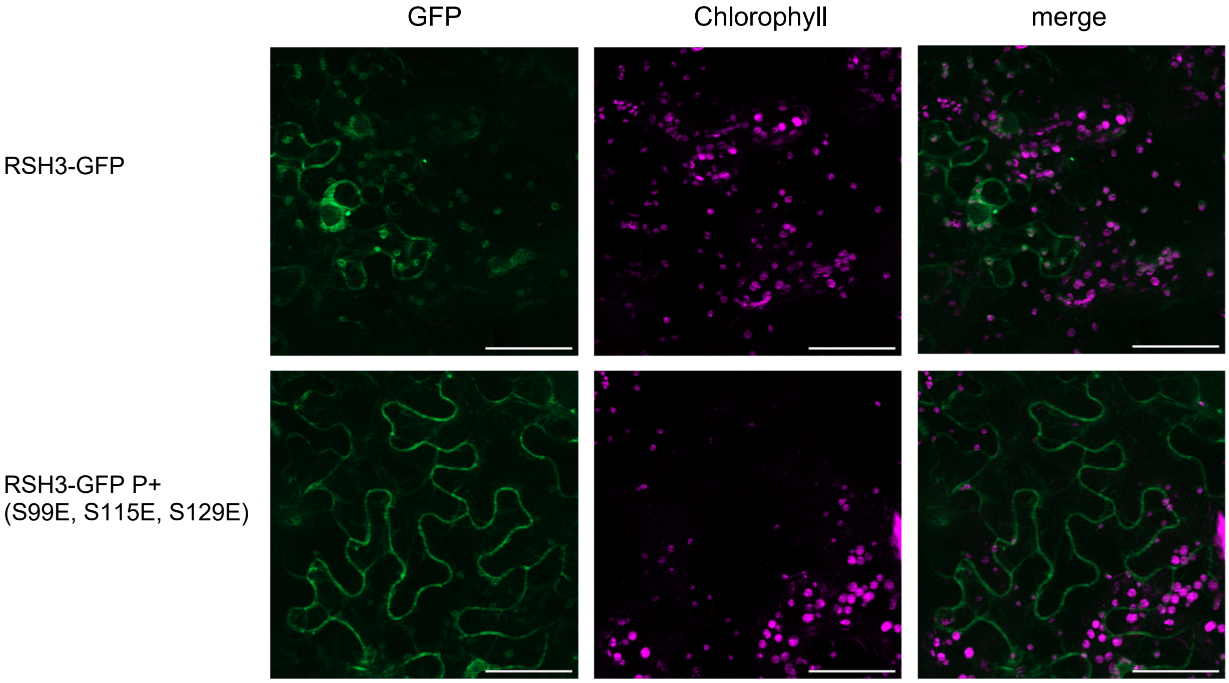

Fig. S12.

The phosphostatus of full length RSH3-GFP influences cellular distribution. Fluorescence microscopy images of *N. benthamiana* expressing RSH3-GFP or RSH3-GFP P+. Scale bar, 50 µm.

Table S1.

List of LST8 interactors identified in a two-hybrid screen in yeast. Performed by Hybrigenics, Paris France with either LexA or Gal4 fusions to LST8 as bait.

Gene accession	Name	Clones	Score*
At1g48970	eIF2B	4	B
**At3g14050**	**RSH2**	**4**	**B**
At3g03790	RCC1	2	C
At3g26890	DUF4210	2	C
**At1g54130**	**RSH3**	**2**	**C**
At5g62000	ARF2	1	D
At4g39140	Ring/U Box	1	D
At4g27030	FAD 4	2	D
At4g18120	Mei2-like 4	1	D
At2g27100	Serrate	1	D
At1g09060	JMJ24	1	E
At5g08720	HCF145	2	F

*A, very high confidence, B, high confidence, C, good confidence, D, moderate confidence and E and F, probably technical artifacts

Table S2.

Arabidopsis plant lines used in this study.

Plant species	Designation	Source or reference	Identifiers	Notes
Arabidopsis thaliana	Col-0	Nottingham *Arabidopsis* Stock Centre (NASC)	WT, Columbia, N1093 (NASC)	
Arabidopsis thaliana	*qrt1-2*	Nottingham *Arabidopsis* Stock Centre (NASC)	N8846 (NASC)	Col-3 ecotype, SAIL insertion parent line
Arabidopsis thaliana	*qrt1-2/rsh1-1*	Nottingham *Arabidopsis* Stock Centre (NASC), (3)	TDNA insertion SAIL_391_E11, N818025 (NASC), *rsh1* mutant	Col-3 ecotype
Arabidopsis thaliana	*qrt1-2/rsh2-1*	Nottingham *Arabidopsis* Stock Centre (NASC), (3)	TDNA insertion SAIL_305_B12, N814119 (NASC), *rsh2* mutant	Col-3 ecotype
Arabidopsis thaliana	*qrt1-2/rsh3-1*	Nottingham *Arabidopsis* Stock Centre (NASC), (3)	TDNA insertion SAIL_99_G05, N862398 (NASC), *rsh3* mutant	Col-3 ecotype
Arabidopsis thaliana	*qrt1-2/rsh2-1 rsh3-1*	Nottingham *Arabidopsis* Stock Centre (NASC), (3)	*rsh2,3* mutant	Col-3 ecotype
Arabidopsis thaliana	*Col-0/OX:RSH1*	(3)	OX:RSH1, OX:RSH1-GFP (10.4)	Genomic sequence of RSH1
Arabidopsis thaliana	*Col-0/OX:RSH3*	(3)	OX:RSH3, OX:RSH3-GFP.1	Genomic sequence of RSH3
Arabidopsis thaliana	*qrt1-2/rsh2-1 rsh3-1 OX:RSH3 line 1*	This study	*rsh2,3* OX:RSH3-GFP line1	Col-3 ecotype, CDS of RSH3
Arabidopsis thaliana	*qrt1-2/rsh2-1 rsh3-1 OX:SSU-RSH3 line 1*	This study	*rsh2,3* OX:SSU-RSH3-GFP line1	Col-3 ecotype, CDS of RSH3
Arabidopsis thaliana	*qrt1-2/rsh2-1 rsh3-1 OX:SSU-RSH3 line 2*	This study	*rsh2,3* OX:SSU-RSH3-GFP line2	Col-3 ecotype, CDS of RSH3
Arabidopsis thaliana	Ws	Nottingham *Arabidopsis* Stock Centre (NASC)	Wild type for *TOR/tor-1*	
Arabidopsis thaliana	*Ws/TOR tor-1*	(57)		T-DNA insertion, TOR GUS translational fusion.
Arabidopsis thaliana	*lst8-1*	(58)	SALK_02459*, lst8-1-1*	Non-viable seeds
Arabidopsis thaliana	*lst8-1 OX:RSH3*	This study	*lst8-1* OX:RSH3, *lst8-1-1* OX:RSH3-GFP.1	Not fertile, Genomic sequence of RSH3

Table S3.

Genetic constructions.

Name	Moclo Level	Notes
pEarleygate 103 35S:RSH3-GFP	NA	(3)
TID	L0	(37)
$RSH3_{1-64}$	L1	Synthesized
$RSH3_{1-130}$ S12A, S40A, S47A, S61A, S99A, S115A, S129A	L1	Synthesized
$RSH3_{1-130}$ S99E, S115E, S129E	L1	Synthesized
LST8 (At3g18140) CDS	L0	cds2ns
$RSH3_{65-715}$ CDS	L0	Synthesized
$RSH3_{65-715}$ D452G CDS	L0	Synthesized
$RSH3_{1-715}$ CDS	L0	cds1ns
$RSH3_{1-715}$ S12A, S40A, S47A, S61A, S99A, S115A, S129A	L0	cds1ns
$RSH3_{1-715}$ S99E, S115E, S129E	L0	cds1ns
Cyanophora $RSH2/3_{90-282}$	L0	Synthesized
Rice $RSH2/3_{34-216}$	L0	Synthesized
Tomato $RSH2/3_{43-226}$	L0	Synthesized
35S:SSU_{1-79}-CFP	L1	(37)
35S:TID-LST8	L1	
35S: LST8-TID	L1	
35S:TID-YFP	L1	
35S:mCHERRY-LST8	L1	
35S: $RSH3_{1-715}$-GFP	L1	
35S: $RSH3_{1-715}$ S12A, S40A, S47A, S61A, S99A, S115A, S129A -GFP	L1	
35S: $RSH3_{1-715}$ S99E, S115E, S129E -GFP	L1	
35S:$RSH3_{39-221}$-GFP	L1	
35S:$RSH3_{39-221}$ S40A, S47A, S61A, S99A, S115A, S129A -GFP	L1	
35S:SSU_{57-79}-$RSH3_{65-221}$-GFP	L1	
35S:SSU-$RSH3_{65-715}$-GFP	L1	
35S:SSU-$RSH3_{194-715}$ D452G-GFP	L1	
35S:SSU_{1-79}-GFP	L1	
35S:GFP	L1	
35S:RSH_{1-70}-GFP	L1	
35S:RSH_{1-96}-GFP	L1	
35S:RSH_{1-135}-GFP	L1	
35S:RSH_{1-165}-GFP	L1	
35S:RSH_{1-175}-GFP	L1	
35S:Cyanophora $RSH2/3_{90-282}$-GFP	L1	
35S:Rice $RSH2/3_{34-216}$-GFP	L1	
35S:Tomato $RSH2/3_{43-226}$-GFP	L1	

Data S1. (separate file)

Data S1 can be found at Dryad (https://doi.org/10.5061/dryad.ghx3ffbtm). Contains underlying data for each figure panel including uncropped blots, sequence data, and analysis scripts.

REFERENCES AND NOTES

1. G. Bange, D. E. Brodersen, A. Liuzzi, W. Steinchen, Two P or not two P: Understanding regulation by the bacterial second messengers (p)ppGpp. *Annu. Rev. Microbiol.* **75**, 383–406 (2021).

2. M. Maekawa, R. Honoki, Y. Ihara, R. Sato, A. Oikawa, Y. Kanno, H. Ohta, M. Seo, K. Saito, S. Masuda, Impact of the plastidial stringent response in plant growth and stress responses. *Nat. Plants* **1**, 1–7 (2015).

3. M. Sugliani, H. Abdelkefi, H. Ke, E. Bouveret, C. Robaglia, S. Caffarri, B. Field, An ancient bacterial signaling pathway regulates chloroplast function to influence growth and development in Arabidopsis. *Plant Cell* **28**, 661–679 (2016).

4. M. Mehrez, S. Romand, B. Field, New perspectives on the molecular mechanisms of stress signalling by the nucleotide guanosine tetraphosphate (ppGpp), an emerging regulator of photosynthesis in plants and algae. *New Phytol.* **237**, 1086–1099 (2023).

5. G. M. Burkart, F. Brandizzi, A tour of TOR complex signaling in plants. *Trends Biochem. Sci.* **46**, 417–428 (2021).

6. J. M. Pacheco, M. V. Canal, C. M. Pereyra, E. Welchen, G. M. A. Martínez-Noël, J. M. Estevez, The tip of the iceberg: Emerging roles of TORC1, and its regulatory functions in plant cells. *J. Exp. Bot.* **72**, 4085–4101 (2021).

7. P. Dong, F. Xiong, Y. Que, K. Wang, L. Yu, Z. Li, R. Maozhi, Expression profiling and functional analysis reveals that TOR is a key player in regulating photosynthesis and phytohormone signaling pathways in Arabidopsis. *Front. Plant Sci.* **6**, 677 (2015).

8. L. Sun, Y. Yu, W. Hu, Q. Min, H. Kang, Y. Li, Y. Hong, X. Wang, Y. Hong, Ribosomal protein S6 kinase1 coordinates with TOR-Raptor2 to regulate thylakoid membrane biosynthesis in rice. *Biochim. Biophys. Acta BBA - Mol. Cell Biol. Lipids* **1861**, 639–649 (2016).

9. S. Imamura, Y. Nomura, T. Takemura, I. Pancha, K. Taki, K. Toguchi, Y. Tozawa, K. Tanaka, The checkpoint kinase TOR (target of rapamycin) regulates expression of a nuclear-encoded chloroplast RelA-SpoT homolog (RSH) and modulates chloroplast ribosomal RNA synthesis in a unicellular red alga. *Plant J.* **94**, 327–339 (2018).

10. S. Upadhyaya, B. J. Rao, Reciprocal regulation of photosynthesis and mitochondrial respiration by TOR kinase in Chlamydomonas reinhardtii. *Plant Direct* **3**, e00184–e00184 (2019).

11. S. D'Alessandro, Coordination of chloroplast activity with plant growth: Clues point to TOR. *Plan. Theory* **11**, 803 (2022).

12. I. Couso, A. L. Smythers, M. M. Ford, J. G. Umen, J. L. Crespo, L. M. Hicks, Inositol polyphosphates and target of rapamycin kinase signalling govern photosystem II protein phosphorylation and photosynthetic function under light stress in Chlamydomonas. *New Phytol.* **232**, 2011–2025 (2021).

13. R. M. Givens, M. H. Lin, D. J. Taylor, U. Mechold, J. O. Berry, V. J. Hernandez, Inducible expression, enzymatic activity, and origin of higher plant homologues of bacterial RelA/SpoT stress proteins in Nicotiana tabacum. *J. Biol. Chem.* **279**, 7495–504 (2004).

14. K. Mizusawa, S. Masuda, H. Ohta, Expression profiling of four RelA/SpoT-like proteins, homologues of bacterial stringent factors, in Arabidopsis thaliana, *Planta* **228**, 553–62 (2008).

15. Y. Zhang, G. Song, N. K. Lal, U. Nagalakshmi, Y. Li, W. Zheng, P. Huang, T. C. Branon, A. Y. Ting, J. W. Walley, S. P. Dinesh-Kumar, TurboID-based proximity labeling reveals that UBR7 is a regulator of N NLR immune receptor-mediated immunity. *Nat. Commun.* **10**, 1–17 (2019).

16. M.-H. Montané, B. Menand, ATP-competitive mTOR kinase inhibitors delay plant growth by triggering early differentiation of meristematic cells but no developmental patterning change. *J. Exp. Bot.* **64**, 4361–4374 (2013).

17. W. V. Bienvenut, D. Sumpton, A. Martinez, S. Lilla, C. Espagne, T. Meinnel, C. Giglione, Comparative large scale characterization of plant versus mammal proteins reveals similar and idiosyncratic N-α-acetylation features. *Mol. Cell. Proteomics* **11**, M111.015131 (2012).

18. P. P. Hsu, S. A. Kang, J. Rameseder, Y. Zhang, K. A. Ottina, D. Lim, T. R. Peterson, Y. Choi, N. S. Gray, M. B. Yaffe, J. A. Marto, D. M. Sabatini, The mTOR-regulated phosphoproteome reveals a mechanism of mTORC1-mediated inhibition of growth factor signaling. *Science* **332**, 1317–1322 (2011).

19. J. Van Leene, C. Han, A. Gadeyne, D. Eeckhout, C. Matthijs, B. Cannoot, N. De Winne, G. Persiau, E. Van De Slijke, B. Van de Cotte, E. Stes, M. Van Bel, V. Storme, F. Impens, K. Gevaert, K. Vandepoele, I. De Smet, G. De Jaeger, Capturing the phosphorylation and protein interaction landscape of the plant TOR kinase. *Nat. Plants* **5**, 316–327 (2019).

20. L. Avilan, C. Puppo, A. Villain, E. Bouveret, B. Menand, B. Field, B. Gontero, RSH enzyme diversity for (p)ppGpp metabolism in Phaeodactylum tricornutum and other diatoms. *Sci. Rep.* **9**, 1–11 (2019).

21. D. Ito, Y. Ihara, H. Nishihara, S. Masuda, Phylogenetic analysis of proteins involved in the stringent response in plant cells. *J. Plant Res.* **130**, 625–634 (2017).

22. J. F. H. Strassert, I. Irisarri, T. A. Williams, F. Burki, A molecular timescale for eukaryote evolution with implications for the origin of red algal-derived plastids. *Nat. Commun.* **12**, 1879 (2021).

23. S. Harchouni, S. England, J. Vieu, S. Romand, A. Aouane, S. Citerne, B. Legeret, J. Alric, Y. Li-Beisson, B. Menand, B. Field, Guanosine tetraphosphate (ppGpp) accumulation inhibits chloroplast gene expression and promotes super grana formation in the moss Physcomitrium (Physcomitrella) patens. *New Phytol.* **236**, 86–98 (2022).

24. L. Avilan, R. Lebrun, C. Puppo, S. Citerne, S. Cuiné, Y. Li-Beisson, B. Menand, B. Field, B. Gontero, ppGpp influences protein protection, growth and photosynthesis in Phaeodactylum tricornutum. *New Phytol.* **230**, 1517–1532 (2021).

25. S. Romand, H. Abdelkefi, C. Lecampion, M. Belaroussi, M. Dussenne, B. Ksas, S. Citerne, J. Caius, S. D'Alessandro, H. Fakhfakh, S. Caffarri, M. Havaux, B. Field, A guanosine tetraphosphate (ppGpp) mediated brake on photosynthesis is required for acclimation to nitrogen limitation in Arabidopsis. *eLife* **11**, e75041 (2022).

26. H. Li, J. Nian, S. Fang, M. Guo, X. Huang, F. Zhang, Q. Wang, J. Zhang, J. Bai, G. Dong, P. Xin, X. Xie, F. Chen, G. Wang, Y. Wang, Q. Qian, J. Zuo, J. Chu, X. Ma, Regulation of nitrogen starvation responses by the alarmone (p)ppGpp in rice. *J. Genet. Genomics* **49**, 469–480 (2022).

27. Y. Liu, X. Duan, X. Zhao, W. Ding, Y. Wang, Y. Xiong, Diverse nitrogen signals activate convergent ROP2-TOR signaling in Arabidopsis. *Dev. Cell* **56**, 1283–1295.e5 (2021).

28. C. Ingargiola, I. Jéhanno, C. Forzani, A. Marmagne, J. Broutin, G. Clément, A.-S. Leprince, C. Meyer, The Arabidopsis target of rapamycin kinase regulates ammonium assimilation and glutamine metabolism. *Plant Physiol.* **192**, 2943–2957 (2023).

29. T. Dobrenel, E. Mancera-Martínez, C. Forzani, M. Azzopardi, M. Davanture, M. Moreau, M. Schepetilnikov, J. Chicher, O. Langella, M. Zivy, C. Robaglia, L. A. Ryabova, J. Hanson, C. Meyer, The Arabidopsis TOR kinase specifically regulates the expression of nuclear genes coding for plastidic ribosomal proteins and the phosphorylation of the cytosolic ribosomal protein S6. *Front. Plant Sci.* **7**, 1611 (2016).

30. M. J. Mallén-Ponce, M. E. Pérez-Pérez, J. L. Crespo, Photosynthetic assimilation of CO_2 regulates TOR activity. *Proc. Natl. Acad. Sci. U.S.A.* **119**, e2115261119 (2022).

31. K. Waegemann, J. Soll, Phosphorylation of the transit sequence of chloroplast precursor proteins. *J. Biol. Chem.* **271**, 6545–6554 (1996).

32. C. Han, W. Hua, J. Li, Y. Qiao, L. Yao, W. Hao, R. Li, M. Fan, G. De Jaeger, W. Yang, M.-Y. Bai, TOR promotes guard cell starch degradation by regulating the activity of β-AMYLASE1 in Arabidopsis. *Plant Cell* **34**, 1038–1053 (2022).

33. N. J. Patron, D. Orzaez, S. Marillonnet, H. Warzecha, C. Matthewman, M. Youles, O. Raitskin, A. Leveau, G. Farré, C. Rogers, A. Smith, J. Hibberd, A. A. R. Webb, J. Locke, S. Schornack, J.

Ajioka, D. C. Baulcombe, C. Zipfel, S. Kamoun, J. D. G. Jones, H. Kuhn, S. Robatzek, H. P. Van Esse, D. Sanders, G. Oldroyd, C. Martin, R. Field, S. O'Connor, S. Fox, B. Wulff, B. Miller, A. Breakspear, G. Radhakrishnan, P.-M. Delaux, D. Loqué, A. Granell, A. Tissier, P. Shih, T. P. Brutnell, W. P. Quick, H. Rischer, P. D. Fraser, A. Aharoni, C. Raines, P. F. South, J.-M. Ané, B. R. Hamberger, J. Langdale, J. Stougaard, H. Bouwmeester, M. Udvardi, J. A. H. Murray, V. Ntoukakis, P. Schäfer, K. Denby, K. J. Edwards, A. Osbourn, J. Haseloff, Standards for plant synthetic biology: A common syntax for exchange of DNA parts. *New Phytol.* **208**, 13–19 (2015).

34. E. Weber, C. Engler, R. Gruetzner, S. Werner, S. Marillonnet, A modular cloning system for standardized assembly of multigene constructs. *PLOS ONE* **6**, e16765 (2011).

35. C. Engler, M. Youles, R. Gruetzner, T.-M. Ehnert, S. Werner, J. D. G. Jones, N. J. Patron, S. Marillonnet, A golden gate modular cloning toolbox for plants. *ACS Synth. Biol.* **3**, 839–843 (2014).

36. J. Gantner, J. Ordon, T. Ilse, C. Kretschmer, R. Gruetzner, C. Löfke, Y. Dagdas, K. Bürstenbinder, S. Marillonnet, J. Stuttmann, Peripheral infrastructure vectors and an extended set of plant parts for the Modular Cloning system. *PLOS ONE* **13**, e0197185 (2018).

37. F. Velay, M. Soula, M. Mehrez, C. Belbachir, S. D'Alessandro, C. Laloi, P. Crete, B. Field, MoBiFC: Development of a modular bimolecular fluorescence complementation toolkit for the analysis of chloroplast protein–protein interactions. *Plant Methods* **18**, 69 (2022).

38. L. Xi, Z. Zhang, W. X. Schulze, "PhosPhAt 4.0: An updated Arabidopsis database for searching phosphorylation sites and kinase-target interactions" in *Plant Phosphoproteomics: Methods and Protocols*, X. N. Wu, Ed. (Springer US, 2021; https://doi.org/10.1007/978-1-0716-1625-3_14) Methods in Molecular Biology, pp. 189–202.

39. C. Chen, R. D. Masi, R. Lintermann, L. Wirthmueller, Nuclear import of Arabidopsis poly(ADP-ribose) polymerase 2 is mediated by importin-α and a nuclear localization sequence located between the predicted SAP domains. *Front. Plant Sci.* **9** (2018).

40. J. Kourelis, F. Kaschani, F. M. Grosse-Holz, F. Homma, M. Kaiser, R. A. L. van der Hoorn, A homology-guided, genome-based proteome for improved proteomics in the alloploid Nicotiana benthamiana. *BMC Genomics* **20**, 722 (2019).

41. Y. Perez-Riverol, J. Bai, C. Bandla, D. García-Seisdedos, S. Hewapathirana, S. Kamatchinathan, D. J. Kundu, A. Prakash, A. Frericks-Zipper, M. Eisenacher, M. Walzer, S. Wang, A. Brazma, J. A. Vizcaíno, The PRIDE database resources in 2022: A hub for mass spectrometry-based proteomics evidences. *Nucleic Acids Res.* **50**, D543–D552 (2022).

42. R. Perdoux, A. Barrada, M. Boulaiz, C. Garau, C. Belbachir, C. Lecampion, M.-H. Montané, B. Menand, A drug-resistant mutation in plant target of rapamycin validates the specificity of ATP-competitive TOR inhibitors in vivo. *Plant J.* **117**, 1344–1355 (2024).

43. G. R. Littlejohn, J. Love, A simple method for imaging Arabidopsis leaves using perfluorodecalin as an infiltrative imaging medium. *J. Vis. Exp* 3394 (2012).

44. C. A. Schneider, W. S. Rasband, K. W. Eliceiri, NIH Image to ImageJ: 25 years of image analysis. *Nat. Methods* **9**, 671–675 (2012).

45. J. Schindelin, I. Arganda-Carreras, E. Frise, V. Kaynig, M. Longair, T. Pietzsch, S. Preibisch, C. Rueden, S. Saalfeld, B. Schmid, J.-Y. Tinevez, D. J. White, V. Hartenstein, K. Eliceiri, P. Tomancak, A. Cardona, Fiji: An open-source platform for biological-image analysis. *Nat. Methods* **9**, 676–682 (2012).

46. J. Bartoli, S. Citerne, G. Mouille, E. Bouveret, B. Field, Quantification of guanosine triphosphate and tetraphosphate in plants and algae using stable isotope-labelled internal standards. *Talanta* **219**, 121261 (2020).

47. K. Katoh, J. Rozewicki, K. D. Yamada, MAFFT online service: Multiple sequence alignment, interactive sequence choice and visualization. *Brief. Bioinform.* **20**, 1160–1166 (2019).

48. J. Trifinopoulos, L.-T. Nguyen, A. von Haeseler, B. Q. Minh, W-IQ-TREE: A fast online phylogenetic tool for maximum likelihood analysis. *Nucleic Acids Res.* **44**, W232–W235 (2016).

49. T. L. Shimada, T. Shimada, I. Hara-Nishimura, A rapid and non-destructive screenable marker, FAST, for identifying transformed seeds of Arabidopsis thaliana. *Plant J. Cell Mol. Biol.* **61**, 519–528 (2010).

50. J. Reback, jbrockmendel, W. McKinney, J. V. den Bossche, M. Roeschke, T. Augspurger, S. Hawkins, P. Cloud, gfyoung, Sinhrks, P. Hoefler, A. Klein, T. Petersen, J. Tratner, C. She, W. Ayd, S. Naveh, J. H. M. Darbyshire, R. Shadrach, M. Garcia, J. Schendel, A. Hayden, D. Saxton, M. E. Gorelli, F. Li, T. Wörtwein, M. Zeitlin, V. Jancauskas, A. McMaster, T. Li, pandas-dev/pandas: Pandas 1.4.3, Zenodo (2022); https://doi.org/10.5281/zenodo.6702671.

51. J. D. Hunter, Matplotlib: A 2D Graphics Environment. *Comput. Sci. Eng.* **9**, 90–95 (2007).

52. M. L. Waskom, seaborn: Statistical data visualization. *J. Open Source Softw.* **6**, 3021 (2021).

53. R. Vallat, Pingouin: Statistics in Python. *J. Open Source Softw.* **3**, 1026 (2018).

54. J. J. Almagro Armenteros, M. Salvatore, O. Emanuelsson, O. Winther, G. von Heijne, A. Elofsson, H. Nielsen, Detecting sequence signals in targeting peptides using deep learning. *Life Sci. Alliance* **2**, e201900429 (2019).

55. R. J. Emenecker, D. Griffith, A. S. Holehouse, Metapredict V2: An update to metapredict, a fast, accurate, and easy-to-use predictor of consensus disorder and structure. bioRxiv [Preprint] (2022). https://doi.org/10.1101/2022.06.06.494887.

56. R. J. Emenecker, D. Griffith, A. S. Holehouse, Metapredict: A fast, accurate, and easy-to-use predictor of consensus disorder and structure. *Biophys. J.* **120**, 4312–4319 (2021).

57. B. Menand, T. Desnos, L. Nussaume, F. Berger, D. Bouchez, C. Meyer, C. Robaglia, Expression and disruption of the Arabidopsis TOR (target of rapamycin) gene. *Proc. Natl. Acad. Sci.* **99**, 6422–6427 (2002).

58. M. Moreau, M. Azzopardi, G. Clément, T. Dobrenel, C. Marchive, C. Renne, M.-L. Martin-Magniette, L. Taconnat, J.-P. Renou, C. Robaglia, C. Meyer, Mutations in the Arabidopsis

homolog of LST8/GβL, a partner of the target of rapamycin kinase, impair plant growth, flowering, and metabolic adaptation to long days. *Plant Cell* **24**, 463–481 (2012).
